# Synthesis and Characterization of Copper(II) and Nickel(II) Complexes with 3-(Morpholin-4-yl)propane-2,3-dione 4-Allylthiosemicarbazone Exploring the Antibacterial, Antifungal and Antiradical Properties

**DOI:** 10.3390/molecules29163903

**Published:** 2024-08-17

**Authors:** Ianina Graur, Vasilii Graur, Marina Cadin, Olga Garbuz, Pavlina Bourosh, Elena Melnic, Carolina Lozan-Tirsu, Greta Balan, Victor Tsapkov, Valeriu Fala, Aurelian Gulea

**Affiliations:** 1 Laboratory of Advanced Materials in Biopharmaceutics and Technics, Institute of Chemistry, Moldova State University, 60 Mateevici Street, MD-2009 Chisinau, Moldova; ulchina.ianina@usm.md (I.G.); victor.tapcov@usm.md (V.T.); guleaaurelian@gmail.com (A.G.); 2 Laboratory of Systematics and Molecular Phylogenetics, Institute of Zoology, Moldova State University, 1 Academiei Street, MD-2028 Chisinau, Moldova; olga.garbuz@sti.usm.md; 3 Institute of Applied Physics, Moldova State University, 5 Academiei Street, MD-2028 Chisinau, Moldova; bourosh.xray@gmail.com (P.B.); elenamelnic2@gmail.com (E.M.); 4 Department of Preventive Medicine, State University of Medicine and Pharmacy “Nicolae Testemitanu”, 165 Stefan cel Mare si Sfant Bd., MD-2004 Chisinau, Moldova; carolina.lozan@usmf.md (C.L.-T.); greta.balan@usmf.md (G.B.); 5 Department of Therapeutic Dentistry, State University of Medicine and Pharmacy “Nicolae Testemitanu”, 165 Stefan cel Mare si Sfant Bd., MD-2004 Chisinau, Moldova; valeriu.fala@usmf.md

**Keywords:** thiosemicarbazone, copper complexes, nickel complexes, antibacterial activity, antifungal activity, antiradical activity

## Abstract

The eleven new copper(II) and nickel(II) coordination compounds [Cu(L)Br]_2_ (**1**), [Cu(L)Cl] (**2**), [Cu(L)NO_3_] (**3**), [Ni(L)Cl] (**4**), [Ni(HL)_2_](NO_3_)_2_ (**5**), and [Cu(A)(L)]NO_3_, where A is 1,10-phenanthroline (**6**), 2,2′-bipyridine (**7**), 3,4-dimethylpyridine (**8**), 3-methylpyridine (**9**), pyridine (**10**) and imidazole (**11**) were synthesized with 3-(morpholin-4-yl)propane-2,3-dione 4-allylthiosemicarbazone (**HL**). The new thiosemicarbazone was characterized by NMR and FTIR spectroscopy. All the coordination compounds were characterized by elemental analysis and FTIR spectroscopy. Also, the crystal structures of **HL** and complexes **1**, **6**, **7**, and **11** were determined using single-crystal X-ray diffraction analysis. Complex **1** has a dimeric molecular structure with two bromide bridging ligands, while **6**, **7**, and **11** are ionic compounds and comprise monomeric complex cations. The studied complexes manifest antibacterial and antifungal activities and also have an antiradical activity that, in many cases, surpasses the activity of trolox, which is used as a standard antioxidant in medicine. Copper complexes **1**–**3** have very weak antiradical properties (IC_50_ > 100 µM), but nickel complexes **4**–**5** are strong antiradicals with IC_50_ values lower than that of trolox. The mixed ligand copper complexes with additional ligand of *N*-heteroaromatic base are superior to complexes without these additional ligands. They are 1.4–5 times more active than trolox.

## 1. Introduction

Thiosemicarbazones are recognized as a significant category of chelating ligands. These ligands show interesting coordination with various transition or non-transition metals. The diverse bonding characteristics and possible uses of thiosemicarbazones and their metal complexes in catalysis [1], electrochemistry [2], as well as biological activities including antimicrobial [3,4], antifungal [5,6], anticancer [7,8], antioxidant [9], and antitubercular [10,11] applications have sparked increased interest in investigating these compounds. Among all the complexes of 3*d* metals with thiosemicarbazones, a special place is occupied by Cu(II) and Ni(II) complexes, which, in addition to exhibiting various types of activities [12,13,14,15,16,17], are also potential antioxidant agents [18,19,20,21,22,23]. Some studies show that introducing an *N*-heteroaromatic base as an additional ligand in the inner sphere of Cu(II) complexes with thiosemicarbazones affects the biological properties of the resulting complexes [24,25].

The modification of the substituent at the fourth position in the composition of thiosemicarbazone often leads to the emergence and increase of its activity and can also affect various physical properties [26]. It was noted that 4-allylthiosemicarbazones show interesting biological activity. The synthesis of these thiosemicarbazones starts from the natural substance allyl isothiocyanate, which exhibits strong antimicrobial activity [27] and anticancer activity in many cancer models [28,29]. Therefore, the presence of a natural substance fragment at the fourth position, which possesses various types of activities, makes the resulting thiosemicarbazone and its complexes potential biologically active substances.

Thiosemicarbazone derivatives of oxoacids also have interesting biological properties. Therefore, they stand out as a separate class for study. The coordination compounds of zinc(II), cobalt, and nickel with thiosemicarbazone of glyoxylic acid effectively inhibit some metabolic enzymes like α-glycosidase, hCA I, hCA II, BChE, and AChE enzymes at the micromolar levels [30,31]. Cu(II) complexes with α-ketoglutaric acid thiosemicarbazone are able to inhibit the cell proliferation of U937 (leukemic derived from histiocytic lymphoma) and induce apoptosis [32]. The thiosemicarbazone derived from pyruvic acid was observed to inhibit both the soluble ornithine carbamoyl transferase in vitro and the enzyme within a cell fraction rich in mitochondria [33]. A Cu(II) coordination compound with pyruvic acid thiosemicarbazone was studied in vivo and showed good activity results against La, P-388, and L-1210 leukemias [34]. In addition, this complex showed antifungal, antimicrobial, and herbicidal activity and was more active than the non-coordinated thiosemicarbazone [24,35].

Previously, we synthesized 3-(piperidin-1-yl)propane-2,3-dione 4-allylthiosemicarbazone to study what effect the introducing of an amide moiety into the thiosemicarbazone of pyruvic acid would have on the structure, physico-chemical properties, and biological activity [36]. Morpholine, which is present in various approved and investigational drugs, as well as bioactive compounds, is a heterocyclic compound. Widely utilized in medicinal chemistry, it is valued for its favorable physicochemical, biological, and metabolic characteristics, along with its easily accessible synthetic pathways [37]. Numerous in vivo investigations have shown that morpholine has the capability not only to enhance potency but also to yield compounds exhibiting favorable drug-like characteristics and improved pharmacokinetics [38,39,40,41]. Thiosemicarbazones that include a morpholine fragment and their complexes have good solubility in water and exhibit various types of biological activity, such as antimicrobial [42] and antiproliferative [43,44] properties, and can be inhibitors of ENPP isozymes [45].

**Figure 1:**
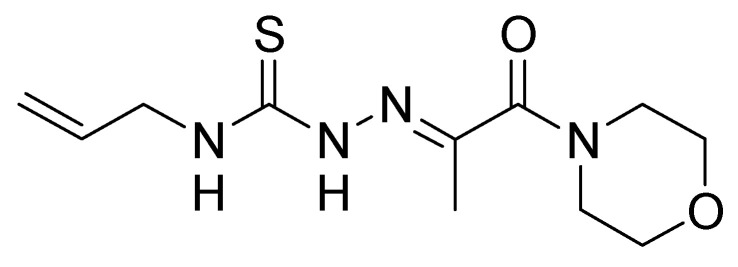
The structural formula of **HL**.

The aim of the present investigation is the synthesis, characterization, and study of antimicrobial, antifungal, and antiradical activities of new Cu(II) and Ni(II) coordination compounds with 3-(morpholin-4-yl)propane-2,3-dione 4-allylthiosemicarbazone (**HL**, Figure 1).

## 2. Results and Discussions

**Scheme 1:**
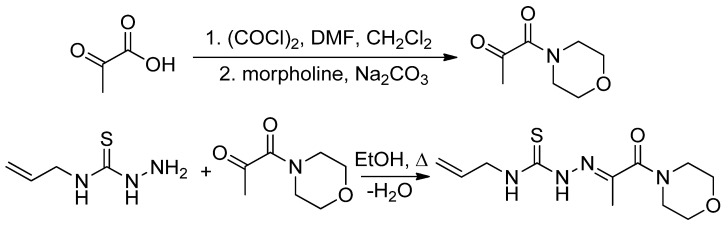
Synthesis of 3-(morpholin-4-yl)propane-2,3-dione 4-allylthiosemicarbazone (**HL**).

In this work, new 3-(morpholin-4-yl)propane-2,3-dione 4-allylthiosemicarbazone (**HL**) was synthesized. It was obtained in two stages. First, pyruvic acid, oxalyl chloride, and morpholine reacted in CH_2_Cl_2_ to obtain 1-(morpholin-4-yl)propane-1,2-dione. In the next step, the reaction between 1-(morpholin-4-yl)propane-1,2-dione and 4-allylthiosemicarbazide in EtOH was performed (Scheme 1).

The structure of the thiosemicarbazone **HL** was confirmed using ^1^H and ^13^C NMR spectroscopy (Figures S1 and S2). The ^1^H NMR spectrum contains multiplets at 5.9, 5.2, and 4.3 ppm, which correspond to allyl fragments in the **HL** molecule. There are also broad singlets at 8.8 and 7.4 ppm, which correspond to the hydrogen atoms of the thiocarbamide NH groups, as well as signals of hydrogen atoms from morpholine moiety in the range of 3.8–3.5 ppm and a signal from the methyl group at 2.1 ppm.

**Scheme 2:**
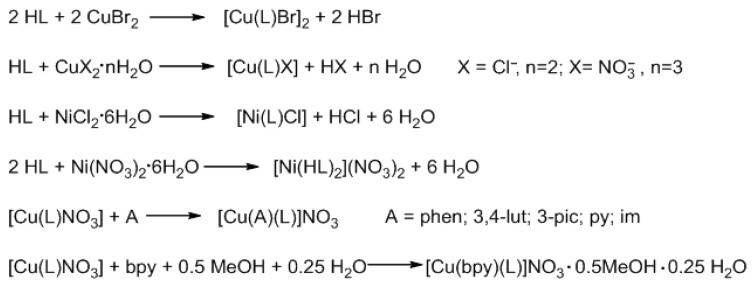
Synthesis of the coordination compounds **1**–**11**.

Eleven new Cu(II) and Ni(II) coordination compounds with **HL** were obtained (Scheme 2). Cu(II) complexes **1**–**3** were synthesized by the interaction of the corresponding Cu(II) salts with thiosemicarbazone **HL** in EtOH in a 1:1 molar ratio. Meanwhile, 1:1 and 1:2 molar ratios between the corresponding Ni(II) salt and **HL** were used for the synthesis of Ni(II) complexes. Cu(II) mixed ligand coordination compounds (**6**–**11**) were obtained by the interaction of complex **3** with the corresponding *N*-heteroaromatic base. The obtained complexes have the following composition: [Cu(L)Br]_2_ (**1**), [Cu(L)Cl] (**2**), [Cu(L)NO_3_] (**3**), [Ni(L)Cl] (**4**), [Ni(HL)_2_](NO_3_)_2_ (**5**), [Cu(phen)(L)]NO_3_ (**6**), [Cu(bpy)(L)]NO_3_·0.5MeOH·0.25H_2_O (**7**), [Cu(3,4-lut)(L)]NO_3_ (**8**), [Cu(3-pic)(L)]NO_3_ (**9**), [Cu(py)(L)]NO_3_ (**10**), [Cu(im)(L)]NO_3_ (**11**) (phen = 1,10-phenanthroline; bpy = 2,2′-bipyridine; 3,4-lut = 3,4-lutidine; 3-pic = 3-picoline; py = pyridine; im = imidazole). They are soluble in organic solvents such as MeOH, EtOH, DMF, and DMSO.

The molar conductivity values of the coordination compounds **1**–**4** and **6**–**11** in MeOH are in the range of 70–98 Ω^−1^∙cm^2^∙mol^−1^, which corresponds to the 1:1 type of electrolyte. The molar conductivity value of the coordination compound **5** is 162 Ω^−1^∙cm^2^∙mol^−1^ which indicates that it behaves as a 1:2 type of electrolyte. This indicates that in the case of complex **5**, there are two nitrate counterions in the outer sphere, while two thiosemicarbazone ligands remain in their non-deprotonated form. Such coordination of non-deprotonated thiosemicarbazones is frequently observed for Ni(II) complexes [23,46].

The FTIR spectra of complexes **1**–**11** (Figures S3–S15) were compared with the spectrum of thiosemicarbazone **HL** to determine the changes that occur during the coordination of the thiosemicarbazone with the central metal cation. It was determined that oxygen, sulfur, and nitrogen donor atoms of **HL** are involved in the process of coordination. In the spectra of complexes **1**–**4** and **6**–**11** the ν(NH) stretching vibration band is shifted by 39–110 cm^−1^ towards higher wavenumbers, while the second ν(NH) absorption band disappears [47,48]. It indicates that thiosemicarbazone **HL** is deprotonated in the process of its coordination to the metal ion. Both absorption bands of ν(NH) groups remain in the FTIR spectrum of Ni(II) complex **5**, indicating that in this complex the thiosemicarbazone **HL** is not deprotonated. Also, small shifts of the absorption bands of ν(C=O) by 8–23 cm^−1^ and of the ν(C=N) by 47–69 cm^−1^ are observed in the FTIR spectra of the synthesized coordination compounds. The absorption band ν(C=S), which was present in the FTIR spectrum of thiosemicarbazone **HL**, disappears in the spectra of the complexes **1**–**4** and **6**–**11**, which also confirms the coordination of thiosemicarbazone in its thiol form. In the spectra of these complexes, a new absorption band ν(C–S) appears in the region of 754–776 cm^−1^. The absorption band ν(C=S) does not disappear in the case of complex **5**. This indicates that **HL** remains in the thione form.

**Figure 2:**
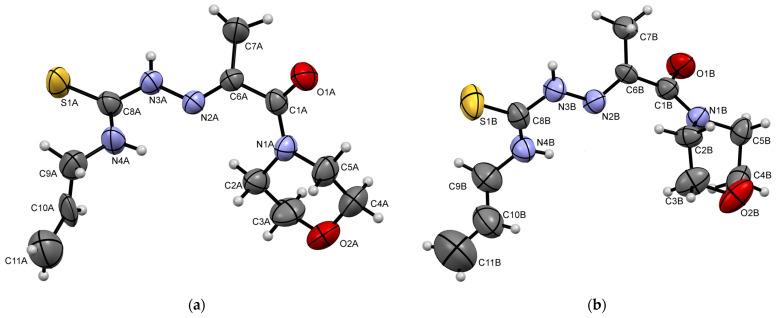
Molecular structures of two crystallographically independent molecules of compound **HL** with numbering scheme; displacement ellipsoids are drawn at the 40% probability. (**a**) molecule A; (**b**) molecule B.

A single crystal X-ray diffraction study of compound **HL** showed that it crystallizes in the *Pbca* orthorhombic space group (Table S1). The asymmetric unit contains two similar molecules A and B (Figure 2). Crystals of related thiosemicarbazone with a piperidine moiety [36] instead of morpholine also crystallize in the *Pbca* orthorhombic space group but with only one molecule in the asymmetric unit cell; two-unit cell parameters are similar to those of crystals of **HL**, but one is about half as long. The structural study revealed that **HL** adopts the same E conformation of the central thiosemicarbazide fragment as in piperidine derivative [36] with torsion angles SCNN and NCNN equal to 174.7 and –9.2° in A and 174.2 and –7.8° in B. The C8–S1, C8–N3, and C8–N4 bond distances are equal to 1.666(5), 1.356(3), and 1.334(5) Å for molecule A and 1.669(5), 1.361(5), and 1.311(6) Å for molecule B, respectively (Table S2) and confirm the stabilization of the ligand in its thione form with the C=S double bond.

**Figure 3:**
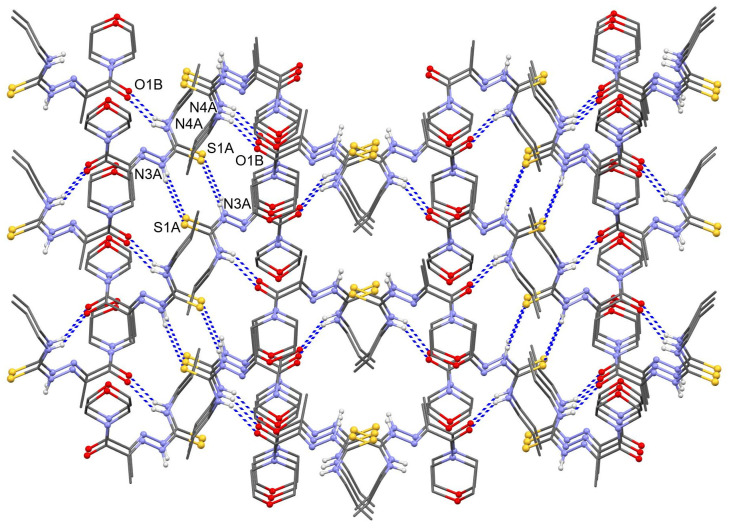
Supramolecular layer forming by H-bonded molecules A and B in the structure of **HL**. H-atoms on carbon atoms are omitted for clarity.

In the crystal, the two crystallographically independent molecules A and B are connected by intermolecular N–H···O hydrogen bonds (Table S3), forming chains in which they alternate (Figure 3), N(4A)···O(1B)/N(4B)···O(1A) 2.830(5)/2.945(5) Å. The inversion-center-related molecules A join each other through two N–H···S hydrogen bonds, forming the *R*^2^_2_(8) graph set and resulting in supramolecular layer formation, N(3A)···S(1A) 3.537(4) Å.

**Figure 4:**
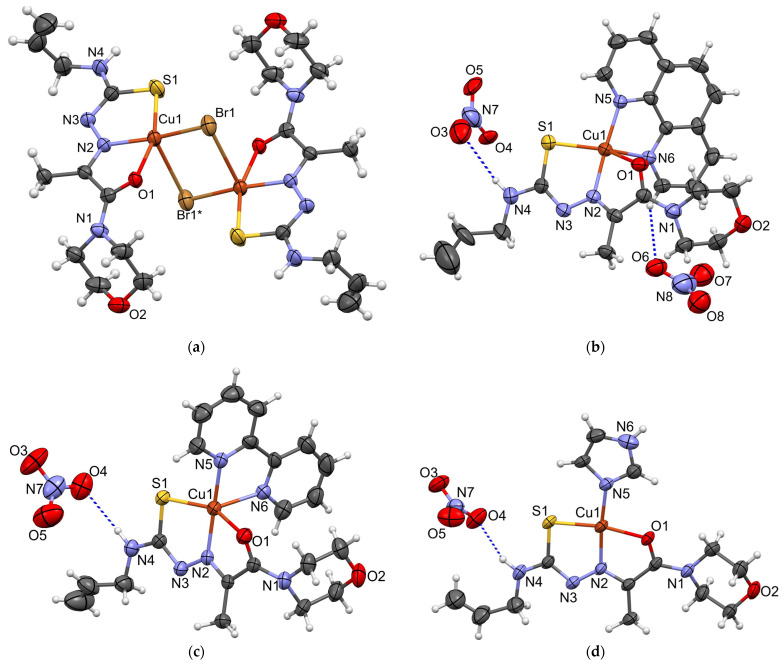
Molecular structure of compounds **1** (**a**), **6** (**b**), **7** (**c**), and **11** (**d**) with a partial numbering scheme; the solvent molecules are omitted.

The Cu(II) complexes **1**, **7** crystallize in the triclinic *P*¯1, **6** in monoclinic *P*2_1_/*n*, and **11** in *P*2_1_/*c* space groups (Table S1). Structural analyses of these compounds revealed that **1** is a centrosymmetric neutral dimeric compound, in which Br(1) and Br(1)* atoms serve as bridging ligands (Figure 4a). The other structurally characterized complexes are ionic, formed by the complex cations [Cu(A)(L)]^+^, where A is phen (in **6**), bpy (in **7**), or imidazole (in **11**), and NO_3_^−^ anions providing charge balance (Figure 4b–d). The asymmetric part of unit cell **6** comprises a cationic mononuclear Cu(II) complex [Cu(phen)(L)]^+^ and two disordered NO_3_^−^ anions occupying special positions on the inversion center. In the crystal of compound **7**, the asymmetric part of the unit cell includes complex cation [Cu(bpy)(L)]^+^ and anion NO_3_^−^ and solvent molecules of MeOH and H_2_O with occupancy coefficients 0.5 and 0.25, respectively. The crystals **11** are built up from [Cu(im)(L)]^+^ cations and NO_3_^−^ anions.

The Cu(1) atom in **1** is penta-coordinated and adopts a square-pyramidal coordination geometry with the ONS set of donor atoms from tridentate monodeprotonated ligand (**L^–^**) and one Br1 atom in basal plane and Br1* atom of center symmetry related monomeric unit in the apex of pyramid, resulting in the dimeric complex formation. The bond distances in the coordination polyhedron Cu(1)–O(1), Cu(1)–N(2), Cu(1)–S(1), Cu(1)–Br(1) and Cu(1)–Br(1)* equal 2.022(3), 1.969(3), 2.235(1), 2.3775(6), and 2.9514(8) Å, respectively (Table S2).

The coordination of the monodeprotonated form of ligand **L^–^** results in its conformational transformation compared with **HL**, similar to that found for the ligand with piperazines [36], namely, the thiosemicarbazone moiety from E to Z form through a rotation around the C8–N3 single bond and additionally by the rotation in around the C(1)–C(6) bond. The tridentate ligand coordinates with the O(1), N(2), and S(1) atoms and forms two conjugated five-membered CuSCNN and CuNCCO metallacycles, with the dihedral angle between their mean planes being 6°. The value of the interatomic distances in the deprotonated thiosemicarbazide fragment highlights a delocalization of the bond lengths (Table S2).

**Figure 5:**
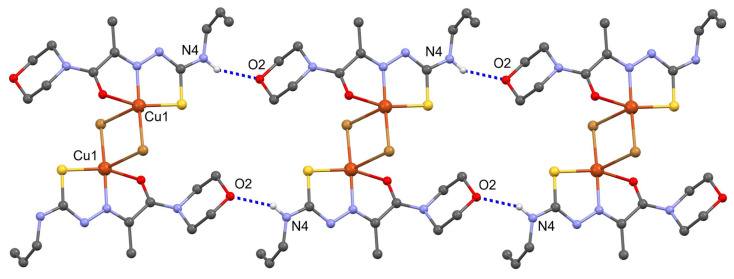
Supramolecular chain in **1**. Non-functional H–atoms are omitted for clarity.

In the crystal, the dimeric compounds join through N4–H∙∙∙O2 hydrogen bonds in supramolecular chains (Figure 5, Table S3), N···O 2.894(5) Å.

The general descriptor τ = (β − α)/60, where α and β are the two largest angles at the center of the metal center, is used to evaluate the degree of distortion the coordination polyhedra in penta-coordinated Cu(1) in **6** and **7**. For the idealized square pyramid and trigonal bipyramid, the extremes are τ = 0 and 1, respectively. In the studied complexes, τ is equal to 0.582 for **6** and 0.352 for **7**, indicating that the N_3_OS surrounding the metal in **6** and **7** is intermediate between square pyramidal and trigonal bipyramidal (Table S2). The monodeprotonated organic ligand **L^–^** coordinates tridentately through the same set of ONS donor atoms as in **1** (Figure 4b,c). The distances Cu(1)–O(1), Cu(1)–N(2), Cu(1)–S(1), Cu(1)–N(5), and Cu(1)–N(6 ) in **6** and **7** are similar, and their medium values are equal to 2.171(4), 1.938(5), 2.249(2), 1.974(4), and 2.132(5) Å, respectively (Table S2).

In complex **11**, unlike **1**, **6**, and **7**, the copper ion displays a square-planar coordination geometry N_2_OS with the average deviation of the coordinated atoms ±0.026 Å from the plane passing through them; the Cu(1) atom is displaced from this plane only by 0.053 Å. The monodeprotonated ligand coordinates to the central atom in the same way as in **1**, **6**, and **7**. The nitrogen atom of the imidazole ligand (Figure 4d) completes the metal surrounding. The interatomic distances Cu(1)–O(1), Cu(1)–N(2), Cu(1)–S(1), and Cu(1)–N(5) are equal to 1.978(3), 1.940(4), 2.232(1), and 1.938(4) Å (Table S2). 

In compounds **6**, **7**, and **11**, the ligand **L^–^** forms, similar to **1**, two conjugated five-membered metallacycles CuSCNN and CuNCCO atoms, the dihedral angle between them being 28.01, 14.61 and 7.09°. As a result, the smaller values for these dihedral angles are set in **1** and **11**, in which the coordination geometry adopts the form of a square-pyramidal or square-planar. The analysis of the interatomic distances in the thiosemicarbazide moiety highlights a delocalization. The value of the C–S bond lengths in these complexes is longer than in **HL**, which indicates a stabilization of the thiol form (Table S2). 

**Figure 6:**
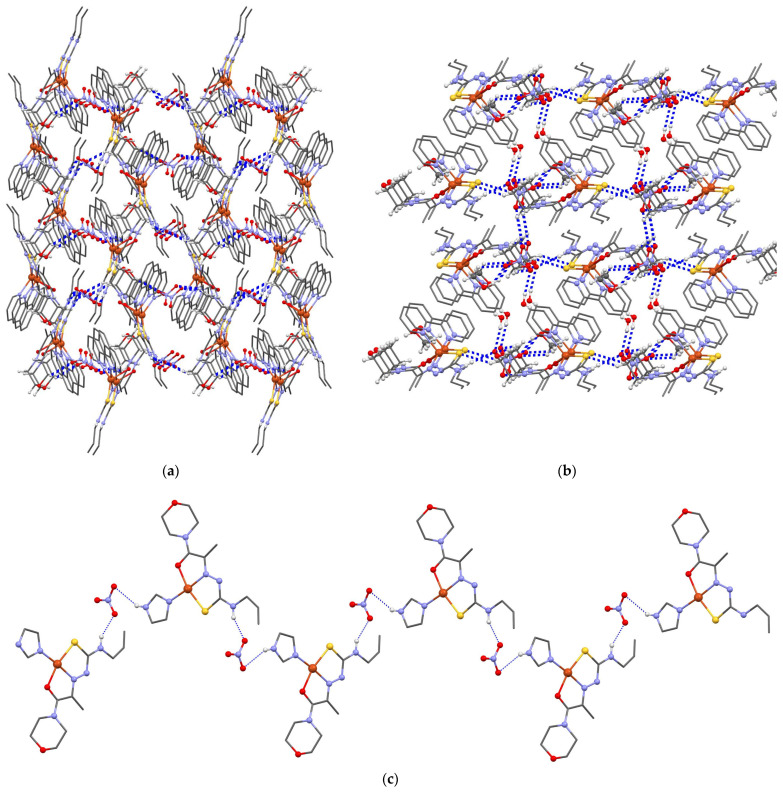
The supramolecular layer in **6** (**a**), the fragment of crystal packing in **7** (**b**), and the supramolecular zigzag-like chain in **11** (**c**). Non-functional H–atoms are omitted for clarity.

In the crystals of compounds **6**, **7**, and **11**, the complex cations [Cu(A)(L)]^+^ are united with NO_3_^−^ anions through N(4)–H·∙∙O hydrogen bonds (Figure 6a–c) producing different supramolecular motifs, N···O 2.96(3)–3.036(6) Å (Table S3). In addition to H bonds in **6** and **7**, the extension of the crystal structures occurs through *π–π* stacking interactions between phen and bpy fragments involving metallacycle. The *π–π* stacking interactions are evidenced by centroid-to-centroid distances in the range of 3.563–3.930 Å. The structure of all complexes is also stabilized by C–H·∙∙O, C–H·∙∙N, C–H·∙∙S, and C–H·∙∙Br hydrogen bonds (Table S3).

The antibacterial and antifungal activities of the **HL** and complexes **1**–**11** were studied on a series of Gram-positive bacteria (*S. aureus*, *B. cereus*), Gram-negative bacteria (*A. baumannii*, *E. coli*), and fungi (*C. albicans*). The obtained results in the form of minimum inhibitory/bactericidal/fungicidal concentrations are shown in Table 1.

Thiosemicarbazone **HL** shows activity only against *B. cereus* and *A. baumannii*, and in other cases, it is not active. Its coordination to the Cu atom leads to an increase in activity mainly against *S. aureus*. The activity of Cu(II) complexes against *B. cereus* is the same as that of **HL** or lower. Among all microorganisms, Cu(II) complexes showed activity only against Gram-positive microorganisms. The coordination of thiosemicarbazone **HL** to the central Ni atom also does not lead to an increase in the activity of the resulting complex **5**. Complexes **6**–**11** with *N*-heteroaromatic bases in the inner sphere are more active than the non-coordinated thiosemicarbazone **HL** and the corresponding complex **3** from which they were synthesized. Some of them are active against a wider range of microorganisms, including Gram-negative microorganisms and fungi. Complex **6** is the most active among all synthesized compounds. The activity of compounds **6**–**11** is influenced by the nature of the *N*-heteroaromatic base in the internal sphere of the complexes. The activity of these compounds decreases according to the following series: phen > 3-pic > py > im > 3,4-lut > bpy. The antifungal activity of complex **6** exceeds the activity of Nystatine and exhibits the same activity as Fluconazole, which is used as a standard antifungal drug.

The antiradical activity against ABTS^•+^ cation radicals was studied for **HL** and complexes **1**–**11**. The obtained results in the form of semimaximal inhibitory concentrations (IC_50_) are shown in Table 2. The coordination of thiosemicarbazone **HL** to the Cu(II) ions leads to a decrease in antiradical activity, while coordination to the Ni atom leads, on the contrary, to an increase in activity. According to the molar conductivity values of Cu(II) complexes **1**–**3**, they dissociate in solution, forming a complex cation and an anion of the corresponding acid residue. The IC_50_ values of these complexes are similar to each other, indicating that while the nature of the acid residue affects the antiradical activity, it is not a major factor for this type of activity. Octahedral Ni(II) complex **5** with two non-deprotonated thiosemicarbazone ligands is more active than square-planar Ni(II) complex **4** with monodeprotonated thiosemicarbazone ligand in the inner sphere. The introduction of all studied *N*-heteroaromatic amines in the inner sphere of Cu(II) complex **3** led to a huge increase in antiradical activity. Almost all of these mixed-ligand complexes are more active than the most active Ni(II) complex (**5**). The activity of complexes **6**–**11** depends on the nature of the *N*-heteroaromatic base in the complex. Complexes containing monodentate amines showed better activity than complexes with bidentate amines (**6**, **7**). Complexes **8** (IC_50_ = 7.3 ± 0.3 μM) and **9** (IC_50_ = 6.7 ± 0.2 μM) are the most active ones. The activity of these complexes exceeds 13–14 times the activity of the thiosemicarbazone **HL** and also exceeds 5 times the activity of Trolox.

**Figure 7:**
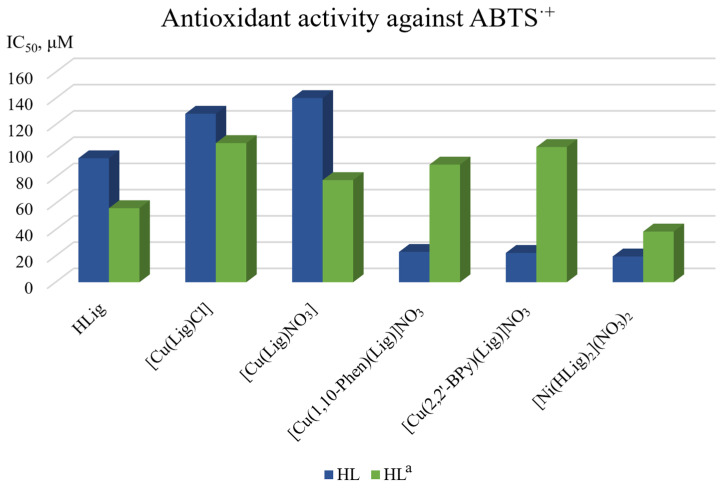
The antiradical activity of some of the studied compounds in comparison with their analogues with **HL^a^** ligand, where HLig is either **HL** (3-(morpholin-4-yl)propane-2,3-dione 4-allylthiosemicarbazone) or **HL^a^** (3-(piperidin-1-yl)propane-2,3-dione 4-allylthiosemicarbazone).

The antiradical activity of the synthesized compounds can be compared with the activity of 3-(piperidin-1-yl)propane-2,3-dione 4-allylthiosemicarbazone (**HL^a^**) and its Cu(II) and Ni(II) complexes that were previously described [36] (Figure 7). Thiosemicarbazone **HL** is less active than its analogue with the piperidine fragment (**HL^a^**). The same is observed in the case of Cu(II) complexes **2** and **3** and their analogues with **HL^a^**. However, the mixed-ligand coordination compounds of Cu(II) **6** and **7** exceed 4–5 times their structural analogues with the **HL^a^** activity of previously published substances similar in structure. The Ni(II) complex **5** is also more active than its structural analogue. This means that the appearance of a morpholine fragment in the composition of thiosemicarbazone and its complexes affects their antiradical activity increasing it in some cases. As it is described in the literature [37], the appearance of morpholine structure changes the polarity and lipophilicity of the resulting compounds, which influences the processes of drug-receptor interactions, as the electronegative oxygen atom in this ring changes the electron density distribution in the molecule.

## 3. Materials and Methods

### 3.1. Materials

All the reagents used were chemically pure. Metal salts CuCl_2_·2H_2_O, CuBr_2_, Cu(NO_3_)_2_·3H_2_O, NiCl_2_·6H_2_O, and Ni(NO_3_)_2_·6H_2_O (Merck, Darmstadt, Germany) and N-heteroaromatic bases (phen, bpy, 3,4-dimethylpyridine, 3-methylpyridine, pyridine, imidazole) were used as supplied. Pyruvic acid, oxalyl chloride, sodium carbonate, dichloromethane, dimethylformamide, allyl isothiocyanate, hydrazine hydrate, and morpholine were used as received (Sigma-Aldrich, Munich, Germany).

4-Allylthiosemicarbazide was prepared similarly to the literature procedure [49] by reaction of the allyl isothiocyanate and a 50–60% (*w*/*w*) aqueous solution of hydrazine. The characteristics of the obtained substance correspond with the data reported in the literature [49].

White solid. Yield: 84%; mp 92–93 °C. FW: 131.20 g/mol; Anal Calc. for C_4_H_9_N_3_S: C, 36.62; H, 6.91; N, 32.03; S, 24.44; found: C, 36.48; H, 6.83; N, 32.09; S, 24.51%. ^1^H NMR (acetone-d_6_, 400 MHz) 9.03 (br s, 1H; NH); 7.86 (br s, 1H; NH); 5.92 (m, 1H; CH); 5.13 (m, 2H; CH_2_); 4.39 (br s, 1H; NH); 4.25 (t, 2H; CH_2_–N). ^13^C NMR (acetone-d_6_, 100 MHz) 178.86 (C=S); 134.76 (CH(_allyl)_); 114.85 (CH_2(allyl,sp_^2^_)_); 45.85 (CH_2(allyl,sp_^3^_)_).

The solvents were purified and dried according to standard procedures [50].

The NMR spectra were recorded on a Bruker DRX-400 using CDCl_3_ as a solvent. FT-IR spectra were recorded on a Bruker ALPHA FTIR spectrophotometer at room temperature in the range of 4000–400 cm^–1^. The elemental analysis was performed similarly to the literature procedures [51] and on the automatic Perkin Elmer 2400 elemental analyzer. The resistance of solutions of complexes in MeOH (20 °C, c 0.001 M) was measured using an R-38 rheochord bridge.

### 3.2. Synthesis

#### 3.2.1. Synthesis of 3-(Morpholin-4-yl)propane-2,3-dione 4-allylthiosemicarbazone

1-(Morpholin-4-yl)propane-1,2-dione

The synthesis was carried out by a similar method described in [36]. Pyruvic acid (8.80 g, 0.100 mol) was dissolved in 5 mL of CH_2_Cl_2_ and placed in a flat-bottomed flask. Oxalyl chloride (15.24 g, 0.120 mol) was dissolved in 10 mL of CH_2_Cl_2_ and added dropwise to the reaction mixture with stirring at 0 °C. Subsequently, three drops of dimethylformamide were introduced as a catalyst, and a condenser fitted with a drying tube containing calcium chloride was attached to the flask. The reaction mixture was stirred and heated for 1.5 h, resulting in the formation of yellow oil. A suspension of morpholine (8.70 g, 0.100 mol), sodium carbonate (10.60 g, 0.100 mol), and 10 mL of CH_2_Cl_2_ was stirred at 0 °C. 2-Oxopropanoyl chloride was added dropwise to the obtained cooled suspension and the mixture was stirred at room temperature for 1 h. After filtration, the mixture was washed with water (3 × 100 cm^3^) and dried in the air to afford the crude product as mobile yellow oil. The characteristics of the obtained substance correspond with the data reported in the literature [52].

Yellow oil. Yield: 40%; FW: 157.17 g/mol; Anal Calc. for C_7_H_11_NO_3_: C, 53.49; H, 7.05; N, 8.91; found: C, 53.57; H, 7.01; N, 8.98. FTIR data (cm^–1^): 1710 (C=O_ketone_); 1638 (C=O_amide_). ^1^H NMR (CDCl_3_, 400 MHz) 3.75–3.54 (m, 4 × 2H; CH_2_); 2.44 (s, 3H; CH_3_). ^13^C NMR (CDCl_3_, 100 MHz) 198.14 (C=O); 164.82 (C=O); 66.78 (CH_2_–O); 46.12 (CH_2_–N); 27.82 (CH_3_).

3-(morpholin-4-yl)propane-2,3-dione 4-allylthiosemicarbazone

1-(Morpholin-4-yl)propane-1,2-dione (3.14 g, 20.0 mmol) reacted with *N*-(prop-2-en-1-yl)hydrazinecarbothioamide (2.62 g, 20.0 mmol) in EtOH with stirring and heating. The obtained pale-yellow precipitate was separated through filtration, washed with a small amount of EtOH, and dried in the air.

Pale yellow solid. Yield: 78%; mp 138–139 °C. FW: 270.35 g/mol; Anal Calc. for C_11_H_18_N_4_O_2_S: C, 48.87; H, 6.71; N, 20.72; S, 11.86; found: C, 48.70; H, 6.61; N, 20.61; S, 11.78%. FTIR data (cm^–1^): ν(N–H) 3282, 3200; ν (C=O) 1636; ν (C=N) 1619; ν (C=S) 1360. ^1^H NMR (CDCl_3_, 400 MHz) 8.75 (br s, 1H; NH); 7.39 (br s, 1H; NH); 5.89 (m, 1H; CH); 5.21 (m, 2H; CH_2_); 4.30 (m, 2H; NH–CH_2_); 3.76–3.56 (m, 4 × 2H; CH_2(morpholine)_); 2.11 (s, 3H; CH_3_). ^13^C NMR (CDCl_3_, 100 MHz) 178.30 (C=S); 165.51 (C=O); 142.32 (C=N); 132.83 (CH(_allyl)_); 117.50 (CH_2(allyl,sp_^2^_)_); 66.72 (CH_2_O_(morpholine)_); 46.95 (CH_2(allyl,sp_^3^_)_ and CH_2_N_(morpholine)_); 14.13 (CH_3_).

#### 3.2.2. Synthesis of Coordination Compounds

[Cu(L)Br]_2_ (**1**) 

Copper(II) bromide (CuBr_2_) (0.224 g, 1 mmol) was added to a hot (55° C) EtOH solution (25 mL) of 3-(morpholin-4-yl)propane-2,3-dione 4-allylthiosemicarbazone **HL** (0.270 g, 1 mmol). The mixture was stirred for 30 min with heating (55 °C). By cooling to room temperature, the green precipitate was obtained. It was filtered out, washed with cold EtOH, and dried in vacuo.

Green solid. Yield: 86%. Anal. Calc. for C_22_H_34_Br_2_Cu_2_N_8_O_4_S_2_ (825.59 g mol^−1^): C, 32.01; H, 4.15; Br, 19.36; Cu, 15.39; N, 13.57; S, 7.77. Found: C, 31.91; H, 4.05; Br, 19.24; Cu, 15.23; N, 13.44; S, 7.65. Main FTIR peaks (cm^−1^): ν(NH) 3288, ν(C=O) 1642, ν(C=N) 1587, ν(C–S) 746. χ(CH_3_OH): 56 Ω^−1^ cm^−2^ mol^−1^.

[Cu(L)Cl] (**2**)

Coordination compound **2** was synthesized similarly to compound **1** using CuCl_2_·2H_2_O (0.171 g; 1 mmol) and **HL** (0.270 g; 1 mmol).

Green solid. Yield: 72%. Anal. Calc. for C_11_H_17_ClCuN_4_O_2_S (368.34 g mol^−1^): C, 35.87; H, 4.65; Cl, 9.63; Cu, 17.25; N, 15.21; S, 8.71. Found: C, 35.77; H, 4.57; Cl, 9.54; Cu, 17.17; N, 15.12; S, 8.64. Main FTIR peaks (cm^−1^): ν(NH) 3310, ν(C=O) 1613, ν(C=N) 1564, ν(C–S) 771. χ(CH_3_OH): 79 Ω^−1^ cm^−2^ mol^−1^.

[Cu(L)NO_3_] (**3**)

Coordination compound **3** was synthesized similarly to compound **1** using Cu(NO_3_)_2_·3H_2_O c and **HL** (0.270 g; 1 mmol).

Green solid. Yield: 78%. Anal. Calc. for C_11_H_17_CuN_5_O_5_S (394.89 g mol^−1^): C, 33.46; H, 4.34; Cu, 16.09; N, 17.73; S, 8.12. Found: C, 33.38; H, 4.26; Cu, 15.93; N, 17.66; S, 8.06. Main FTIR peaks (cm^−1^): ν(NH) 3298, ν(C=O) 1619, ν(C=N) 1583, ν(C–S) 772. χ(CH_3_OH): 72 Ω^−1^ cm^−2^ mol^−1^.

[Ni(L)Cl] (**4**)

Coordination compound **4** was synthesized similarly to compound **1** using NiCl_2_·6H_2_O (0.238 g; 1 mmol) and **HL** (0.270 g; 1 mmol).

Red solid. Yield: 82%. Anal. Calc. for C_11_H_17_ClN_4_NiO_2_S (363.49 g mol^−1^): C, 36.35; H, 4.71; Cl, 9.75; N, 15.41; Ni, 16.15; S, 8.82. Found: C, 36.25; H, 4.66; Cl, 9.65; N, 15.33; Ni, 16.09; S, 8.77. Main FTIR peaks (cm^−1^): ν(NH) 3251, ν(C=O) 1623, ν(C=N) 1568, ν(C–S) 754. χ(CH_3_OH): 73 Ω^−1^ cm^−2^ mol^−1^.

[Ni(HL)_2_](NO_3_)_2_ (**5**)

Coordination compound **5** was synthesized similarly to compound **1** using Ni(NO_3_)_2_·6H_2_O (0.291 g; 1 mmol) and **HL** (0.540 g; 2 mmol).

Green solid. Yield: 76%. Anal. Calc. for C_22_H_36_N_10_NiO_10_S_2_ (723.41 g mol^−1^): C, 36.53; H, 5.02; N, 19.36; Ni, 8.11; S, 8.87. Found: C, 36.46; H, 4.94; N, 19.30; Ni, 8.07; S, 8.78. Main FTIR peaks (cm^−1^): ν(NH) 3280, 3200, ν(C=O) 1625, ν(C=N) 1570, ν(C=S) 1403. χ(CH_3_OH): 162 Ω^−1^ cm^−2^ mol^−1^.

[Cu(phen)(L)]NO_3_ (**6**)

Phen (0.180 g; 1 mmol) was added to a hot (55 °C) EtOH solution (25 mL) of the coordination compound **3** (0.395 g; 1 mmol). The mixture was stirred for 30 min with heating (55 °C). By cooling to room temperature, the green precipitate was obtained. It was filtered out, washed with cold EtOH, and dried in vacuo.

Green solid. Yield: 82%. Anal. Calc. for C_23_H_25_CuN_7_O_5_S (575.10 g mol^−1^): C, 48.03; H, 4.38; Cu, 11.05; N, 17.05; S, 5.58. Found: C, 47.94; H, 4.30; Cu, 11.00; N, 16.93; S, 5.48. Main FTIR peaks (cm^−1^): ν(NH) 3239, ν(C=O) 1625, ν(C=N) 1556, ν(C–S) 769. χ(CH_3_OH): 98 Ω^−1^ cm^−2^ mol^−1^.

[Cu(bpy)(L)]NO_3_·0.5MeOH·0.25H_2_O (**7**)

Bpy (0.156 g; 1 mmol) was added to a hot (55 °C) MeOH solution (25 mL) of the coordination compound **3** (0.395 g; 1 mmol). The mixture was stirred for 30 min with heating (55 °C). By cooling to room temperature, the green precipitate was obtained. It was filtered out, washed with cold EtOH, and dried in vacuo.

Green solid. Yield: 89%. Anal. Calc. for C_21_._5_H_27_._5_CuN_7_O_5_._75_S (571.60 g mol^−1^): C, 45.18; H, 4.85; Cu, 11.12; N, 17.15; S, 5.61. Found: C, 45.30; H, 4.79; Cu, 11.17; N, 17.29; S, 5.67. Main FTIR peaks (cm^−1^): ν(NH) 3286, ν(C=O) 1625, ν(C=N) 1552, ν(C–S) 767. χ(CH_3_OH): 60 Ω^−1^ cm^−2^ mol^−1^.

[Cu(3,4-lut)(L)]NO_3_ (**8**)

Coordination compound **8** was synthesized similarly to compound **6** using 3,4-lutidine (0.107 g; 1 mmol) and **3** (0.395 g; 1 mmol).

Green solid. Yield: 75%. Anal. Calc. for C_18_H_26_CuN_6_O_5_S (502.05 g mol^−1^): C, 43.06; H, 5.22; Cu, 12.66; N, 16.74; S, 6.39. Found: C, 42.98; H, 5.18; Cu, 12.60; N, 16.68; S, 6.31. Main FTIR peaks (cm^−1^): ν(NH) 3266, ν(C=O) 1613, ν(C=N) 1552, ν(C–S) 767. χ(CH_3_OH): 82 Ω^−1^ cm^−2^ mol^−1^.

[Cu(3-pic)(L)]NO_3_ (**9**)

Coordination compound **9** was synthesized similarly to compound **6** using 3-picoline (0.093 g; 1 mmol) and **3** (0.395 g; 1 mmol).

Green solid. Yield: 79%. Anal. Calc. for C_17_H_24_CuN_6_O_5_S (488.02 g mol^−1^): C, 41.84; H, 4.96; Cu, 13.02; N, 17.22; S, 6.57. Found: C, 41.77; H, 4.87; Cu, 12.94; N, 17.17; S, 6.49. Main FTIR peaks (cm^−1^): ν(NH) 3294, ν(C=O) 1644, ν(C=N) 1556, ν(C–S) 776. χ(CH_3_OH): 98 Ω^−1^ cm^−2^ mol^−1^.

[Cu(py)(L)]NO_3_ (**10**)

Coordination compound **10** was synthesized similarly to compound **6** using pyridine (0.079 g; 1 mmol) and **3** (0.395 g; 1 mmol).

Green solid. Yield: 84%. Anal. Calc. for C_16_H_22_CuN_6_O_5_S (473.99 g mol^−1^): C, 40.54; H, 4.68; Cu, 13.41; N, 17.73; S, 6.76. Found: C, 40.46; H, 4.60; Cu, 13.34; N, 17.65; S, 6.65. Main FTIR peaks (cm^−1^): ν(NH) 3241, ν(C=O) 1640, ν(C=N) 1555, ν(C–S) 773. χ(CH_3_OH): 87 Ω^−1^ cm^−2^ mol^−1^.

[Cu(im)(L)]NO_3_ (**11**)

Coordination compound **11** was synthesized similarly to compound **6** using imidazole (0.068 g; 1 mmol) and **3** (0.395 g; 1 mmol).

Green solid. Yield: 83%. Anal. Calc. for C_14_H_21_CuN_7_O_5_S (462.98 g mol^−1^): C, 36.32; H, 4.57; Cu, 13.73; N, 21.18; S, 6.93. Found: C, 36.25; H, 4.50; Cu, 13.65; N, 21.10; S, 6.86. Main FTIR peaks (cm^−1^): ν(NH) 3244, ν(C=O) 1619, ν(C=N) 1550, ν(C–S) 756. χ(CH_3_OH): 82 Ω^−1^ cm^−2^ mol^−1^.

**Figure 8:**
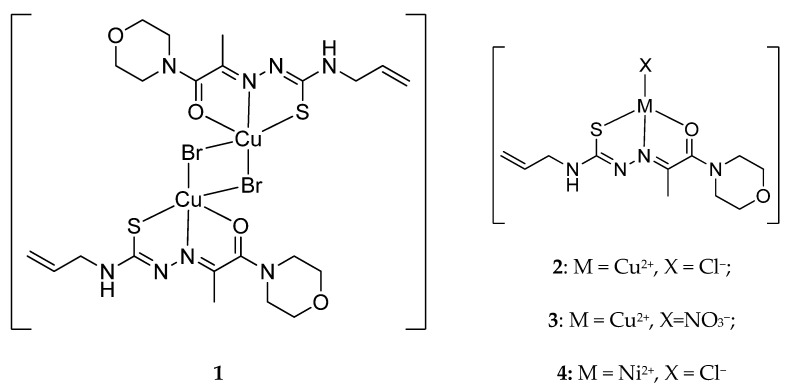

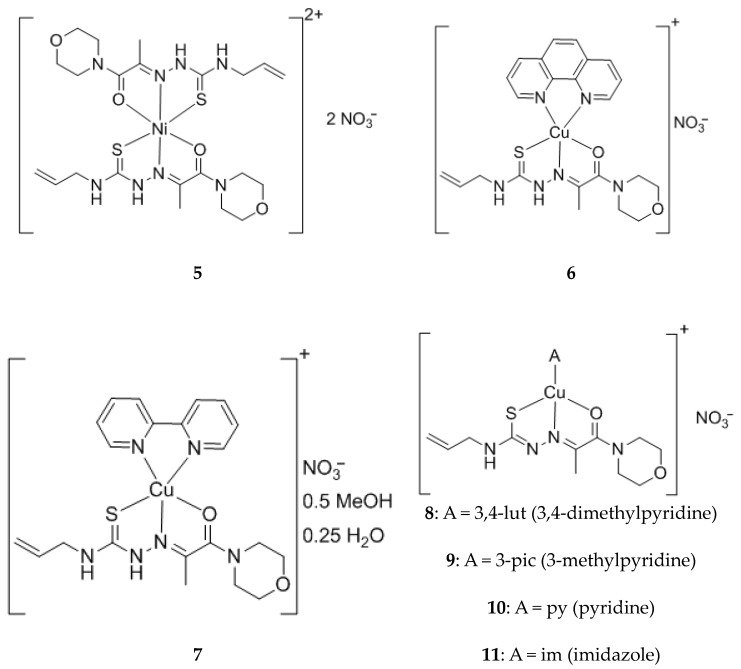
Proposed chemical structures of complexes **1**–**11**.

The proposed chemical structures are represented in Figure 8.

### 3.3. X-ray Crystallography

The X-ray diffraction measurements of crystals of **HL** and complexes **1**, **6**, **7**, and **11** were performed on an Xcalibur E diffractometer equipped with a charge-coupled device (CCD) area detector and a graphite monochromator utilizing MoKα radiation (0.71073 Å) at room temperature (293 K). Data collection and reduction and unit cell determination were obtained using CrysAlis PRO CCD (Oxford Diffraction) [53]. The SHELXS97 and SHELXL2014 program packages [54,55] were used to solve and refine the structures. The structures were solved by direct methods and refined by full-matrix least-squares on F2 with anisotropic displacement parameters for all nonhydrogen atoms. The C-bound H–atoms were positioned geometrically and treated as riding atoms using SHELXL default parameters with U_iso_(H) = 1.2U_eq_(C) and U_iso_(H) = 1.5U_eq_(CH_3_). The disordering problems were resolved for morpholine and allyl fragments in **HL** and for NO_3_^−^ anions, which are disordered over two positions in **6.**

The X-ray data and details of the refinement for **HL** and complexes **1**, **6**, **7**, and **11** are summarized in Table S1. The selected bond distances and angles in **HL** and complexes **1**, **6**, **7**, and **11** have common values and are summarized in Table S2. The figures were produced using MERCURY [56].

### 3.4. Antibacterial and Antifungal Activity

Standard strains of *Staphylococcus aureus* (ATCC 25923), *Bacillus cereus* (ATCC 11778), *Acinetobacter baumannii* (BAA-747), *Escherichia coli* (ATCC 25922), and *Candida albicans* (ATCC 10231) were used to study antibacterial and antifungal activities of the **HL** and coordination compounds **1**–**11**. The minimum inhibitory concentrations (MICs, μg mL^−1^), minimum bactericidal concentrations (MBCs, μg mL^−1^), and minimum fungicidal concentrations (MFCs, μg mL^−1^) were determined using the method of serial dilutions in liquid broth. The tested substances were dissolved in DMSO at a concentration of 10 mg mL^−1^. The next dilutions were made using 2% of peptone bullion. Furacillinum was used as the standard antibacterial drug, whereas Nystatine and Fluconazole were used as the standard antifungal drugs.

### 3.5. Antiradical Activity

The antiradical activity of the synthesized compounds was studied using the ABTS^•+^ method described in [57,58] with modifications. The ABTS^•+^ radical was generated by reacting a 7 mM solution of ABTS (2,2′-azino-bis(3-ethylbenzothiazoline-6-sulphonic acid)) from Sigma with a 2.45 mM solution of potassium persulfate (K_2_S_2_O_8_), also from Sigma. This reaction took place at 25 °C in the dark for 12–20 h at room temperature. Subsequently, the resulting solution was diluted by combining it with acetate-buffered saline (0.02 M, pH 6.5) to achieve an absorbance of 0.70 ± 0.01 units at 734 nm. Dilutions of the studied compounds were prepared in DMSO, with concentrations ranging from 1 to 100 μM. Subsequently, 20 μL of each compound’s dilution was transferred to a 96-well microtiter plate, and 180 μL of the ABTS^•+^ working solution was dispensed using the dispense module of the hybrid reader (BioTek). The reduction in absorbance at 734 nm was precisely measured after a 30-min incubation at 25 °C. All measurements were conducted in triplicate. DMSO served as the negative control and blank samples were run using solvent without ABTS^•+^. The hybrid reader used for the measurement was the Synergy H1 from BioTek. All experiments were conducted three times, and the results obtained were averaged.

## 4. Conclusions

The new 3-(morpholin-4-yl)propane-2,3-dione 4-allylthiosemicarbazone **HL** and 11 new Cu(II) and Ni(II) coordination compounds have been synthesized. The study of the biological activity of the synthesized substances showed that they exhibit antimicrobial, antifungal, and antiradical activity. Cu(II) complexes with *N*-heteroaromatic bases in the internal sphere proved to be the most active; they also exhibited superior activity compared to the medicinal drugs used as standards. Complex **6** exceeds the activity of Nystatine and exhibits the same activity as Fluconazole. Regarding antiradical activity, complex **9** showed the highest activity with an IC_50_ value of 6.7 μM. This study confirms that thiosemicarbazones and their complexes represent a promising avenue in the biomedical field. It is possible to influence their biological activity by altering their structure.

## Figures and Tables

**Table 1:** Antibacterial and antifungal activities of **HL** and complexes **1**–**11** as MIC/MBC/MFC values in μg mL^−1^.

Compound	*Staphylococcus aureus* ATCC 25923		*Bacillus cereus* ATCC 11778		*Acinetobacter baumannii* BAA-747		*Escherichia coli* ATCC 25922		*Candida albicans* ATCC 10231	
	MIC	MBC	MIC	MBC	MIC	MBC	MIC	MBC	MIC	MFC
**HL**	-	-	62.5	62.5	250	500	-	-	-	-
**1**	125	125	62.5	62.5	-	-	-	-	-	-
**2**	250	250	125	125	500	-	-	-	-	-
**3**	62.5	125	125	125	-	-	-	-	500	500
**5**	500	-	125	125	-	-	-	-	-	-
**6**	31.3	31.3	15.6	15.6	125	125	250	250	15.6	31.3
**7**	250	250	250	250	500	500	-	-	62.50	250
**8**	125	125	62.50	125	-	-	-	-	500	-
**9**	31.3	31.3	15.6	31.3	-	-	-	-	250	500
**10**	31.3	125	15.6	31.3	-	-	500	500	-	-
**11**	62.5	62.5	62.5	62.5	-	-	-	-	250	500
Furacillinum	9.3	9.3	4.7	4.7	18.5	37.5	4.7	9.4	-	-
Nystatine	-	-	-	-	-	-	-	-	80	80
Fluconazole	-	-	-	-	-	-	-	-	15.6	31.3

Note: MIC—minimum inhibitory concentration; MBC—minimum bactericidal concentration; MFC—minimum fungicidal concentration; “-”—not active.

**Table 2:** Antiradical activity of **HL** and complexes **1**–**11** against cation radicals ABTS^•+^.

Compound	**HL**	**1**	**2**	**3**	**4**	**5**	**6**
IC_50_, μM	94.4 ± 4.9	168.5 ± 5.0	128.4 ± 3.5	140.3 ± 3.5	30.7 ± 0.8	19.6 ± 0.1	23.0 ± 0.3
Compound	**7**	**8**	**9**	**10**	**11**	Trolox	
IC_50_, μM	22.3 ± 0.3	7.3 ± 0.3	6.7 ± 0.2	17.1 ± 0.7	11.4 ± 0.4	33.3 ± 0.2	

## Data Availability

Data is contained within the article or Supplementary Material.

## Supplementary Materials

The following supporting information can be downloaded at: https://www.mdpi.com/article/10.3390/molecules29163903/s1, Figure S1: ^1^H NMR spectrum of 3-(morpholin-4-yl)propane-2,3-dione 4-allylthiosemicarbazone (**HL**); Figure S2: ^13^C NMR spectrum of 3-(morpholin-4-yl)propane-2,3-dione 4-allylthiosemicarbazone (**HL**); Figure S3: FTIR spectrum of 1-(morpholin-4-yl)propane-1,2-dione; Figure S4: FTIR spectrum of **HL**; Figures S5–S15: FTIR spectra of **1–11**; Table S1: Crystal and Structure Refinement Date for **HL** and **1**, **6**, **7**, and **11**; Table S2: (**a**) Bond Lengths (Å) and Angles (deg) in Coordination Metal Environment in **1**, **6**, **7**, and **11**; (**b**) Selected Bond Lengths (Å) and Angles (deg) in ligands in **HL**, **1**, **6**, **7**, and **11**; Table S3: Hydrogen Bond Distances (Å) and Angles (deg) for **HL** and **1**, **6**, **7**, and **11**. The crystallographic data for compounds **HL**, **1**, **6**, **7**, and **11** (CCDC 2350142–2350146) were deposited with the Cambridge Crystallographic Data Centre. Copies of this information may be obtained free of charge from the Director, CCDC, 12 Union Road, Cambridge CHB2 1EZ, UK (Fax: +44-1223-336033; e-mail: deposit@ccdc.cam.ac.uk or www.ccdc.cam.ac.uk).
