# Supplementary material for: Synthesis and Characterization of Copper(II) and Nickel(II) Complexes with 3-(Morpholin-4-yl)propane-2,3-dione 4-Allylthiosemicarbazone Exploring the Antibacterial, Antifungal and Antiradical Properties
Source: Molecules. 2024 Aug 17;29(16):3903. doi: 10.3390/molecules29163903 (PMC11356811; doi:10.3390/molecules29163903)
Supplement: Supplementary file 1 [file molecules-29-03903-s001.zip › molecules-3070730-supplementary.pdf]

Synthesis and Characterization of Copper(II) and Nickel(II) Complexes with 3-(Morpholin-4-yl)propane-2,3-dione 4-Allylthiosemicarbazone exploring the Antibacterial, Antifungal and Antiradical Properties

Ianina Graur [1], Vasilii Graur [1,*], Marina Cadin [1], Olga Garbuz [2], Pavlina Bourosh [3], Elena Melnic [3], Carolina Lozan-Tirsu [4], Greta Balan [4], Victor Tsapkov [1], Valeriu Fala [5] and Aurelian Gulea [1]

[1] Laboratory of Advanced Materials in Biopharmaceutics and Technics, Institute of Chemistry, Moldova State University, 60 Mateevici Street, MD-2009 Chisinau, Moldova; ulchina.ianina@usm.md (I.G.); victor.tapcov@usm.md (V.T.); guleaaurelian@gmail.com (A.G.)

[2] Laboratory of Systematics and Molecular Phylogenetics, Institute of Zoology, Moldova State University, 1 Academiei Street, MD-2028 Chisinau, Moldova; olga.garbuz@sti.usm.md

[3] Institute of Applied Physics, Moldova State University, 5 Academiei Street, MD-2028 Chisinau, Moldova; bourosh.xray@gmail.com (P.B.); elenamelnic2@gmail.com (E.M.)

[4] Department of Preventive Medicine, State University of Medicine and Pharmacy "Nicolae Testemitanu", 165 Stefan cel Mare si Sfant Bd., MD-2004 Chisinau, Moldova; carolina.lozan@usmf.md (C.L.-T.); greta.balan@usmf.md (G.B.)

[5] Department of Therapeutic Dentistry, State University of Medicine and Pharmacy "Nicolae Testemitanu", 165 Stefan cel Mare si Sfant Bd., MD-2004 Chisinau, Moldova; valeriu.fala@usmf.md

* Correspondence: vasilii.graur@usm.md; Tel.: +373-79389792

Content

Figure S1. ^{1}H NMR (400 MHz, $CDCl_3$) spectrum of 3-(morpholin-4-yl)propane-2,3-dione 4-allylthiosemicarbazone (**HL**). 2

Figure S2. ^{13}C NMR (400 MHz, $CDCl_3$) spectrum of 3-(morpholin-4-yl)propane-2,3-dione 4-allylthiosemicarbazone (**HL**). 3

Figure S3. FTIR spectrum of 1-(morpholin-4-yl)propane-1,2-dione....3

Figure S4. FTIR spectrum of **HL**. 3

Figure S5. FTIR spectrum of **1**. 4

Figure S6. FTIR spectrum of **2**. 4

Figure S7. FTIR spectrum of **3**. 5

Figure S8. FTIR spectrum of **4**. 5

Figure S9. FTIR spectrum of **5**. 6

Figure S10. FTIR spectrum of **6**. 6

Figure S11. FTIR spectrum of **7**. 7

Figure S12. FTIR spectrum of **8**. 7

Figure S13. FTIR spectrum of **9**. 8

Figure S14. FTIR spectrum of **10**. 8

Figure S15. FTIR spectrum of **11**. 9

Table S1. Crystal and Structure Refinement Date for **HL** and **1, 6, 7,** and **11** 9

Table S2. **a)** Bond Lengths (Å) and Angles (deg) in Coordination Metal Environment in **1**, **6**, **7**, and **11**........10

Table S3. Hydrogen Bond Distances (Å) and Angles (deg) for **HL** and **1**, **6**, **7**, and **11**..........11

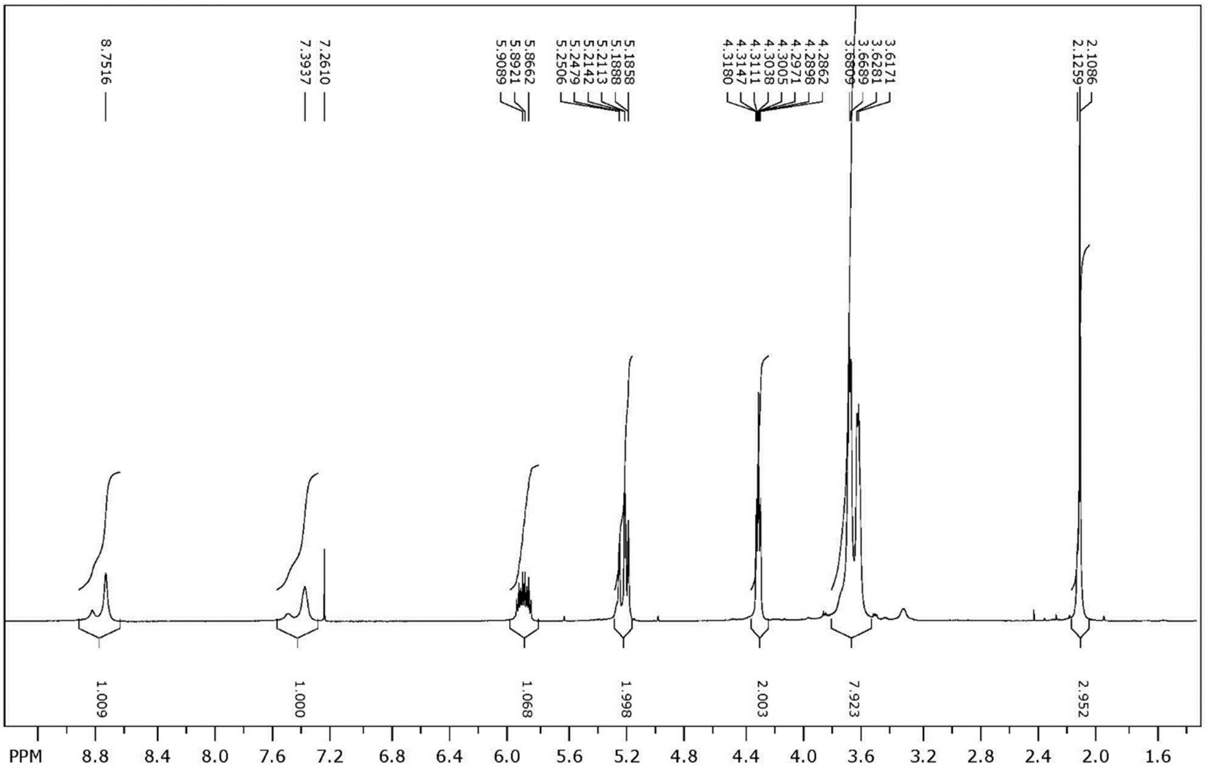

Figure S1. ^1H NMR (400 MHz, $CDCl_3$) spectrum of 3-(morpholin-4-yl)propane-2,3-dione 4-allylthiosemicarbazone (**HL**).

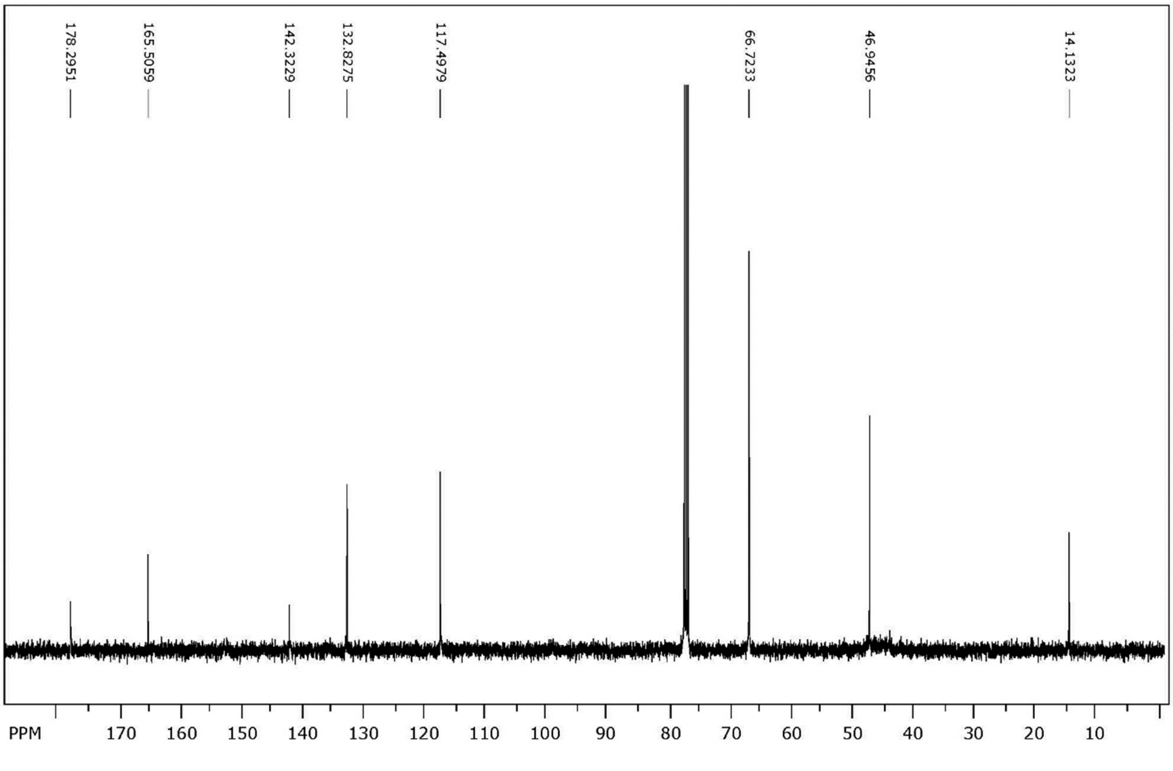

Figure S2. ^{13}C NMR (400 MHz, $CDCl_3$) spectrum of 3-(morpholin-4-yl)propane-2,3-dione 4-allylthiosemicarbazone (**HL**).

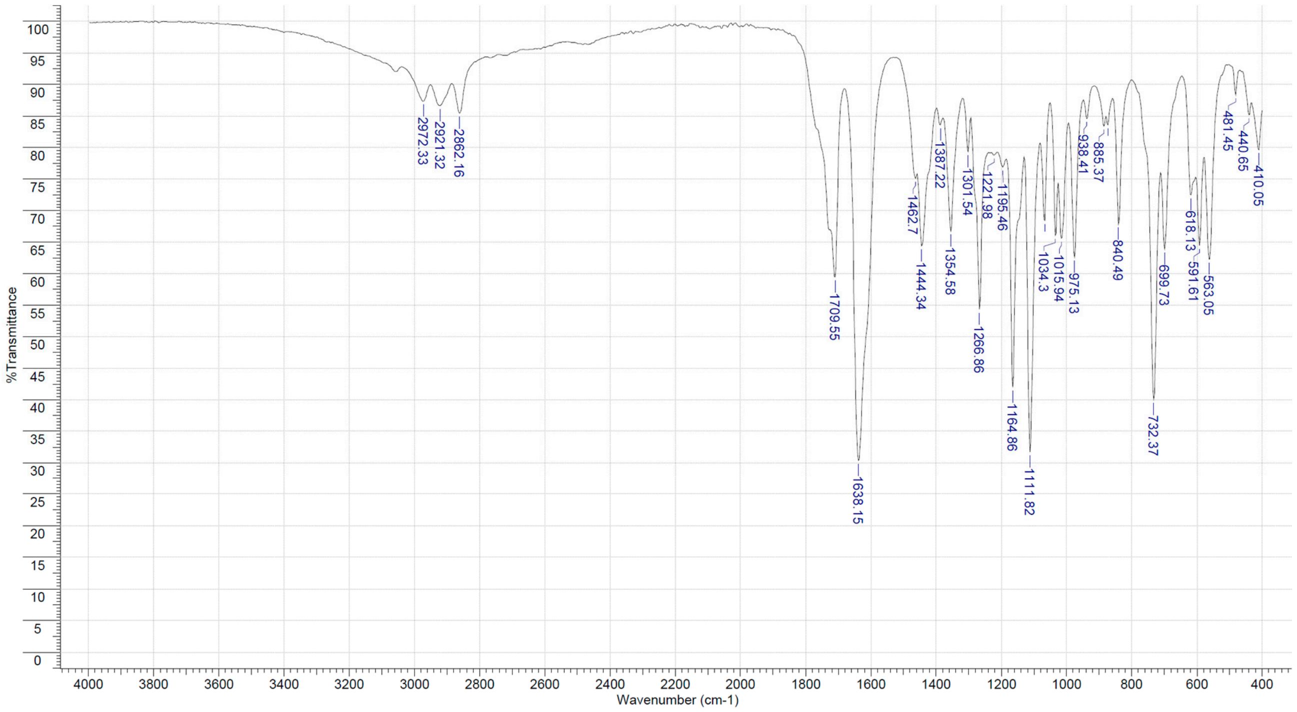

Figure S3. FTIR spectrum of 1-(morpholin-4-yl)propane-1,2-dione.

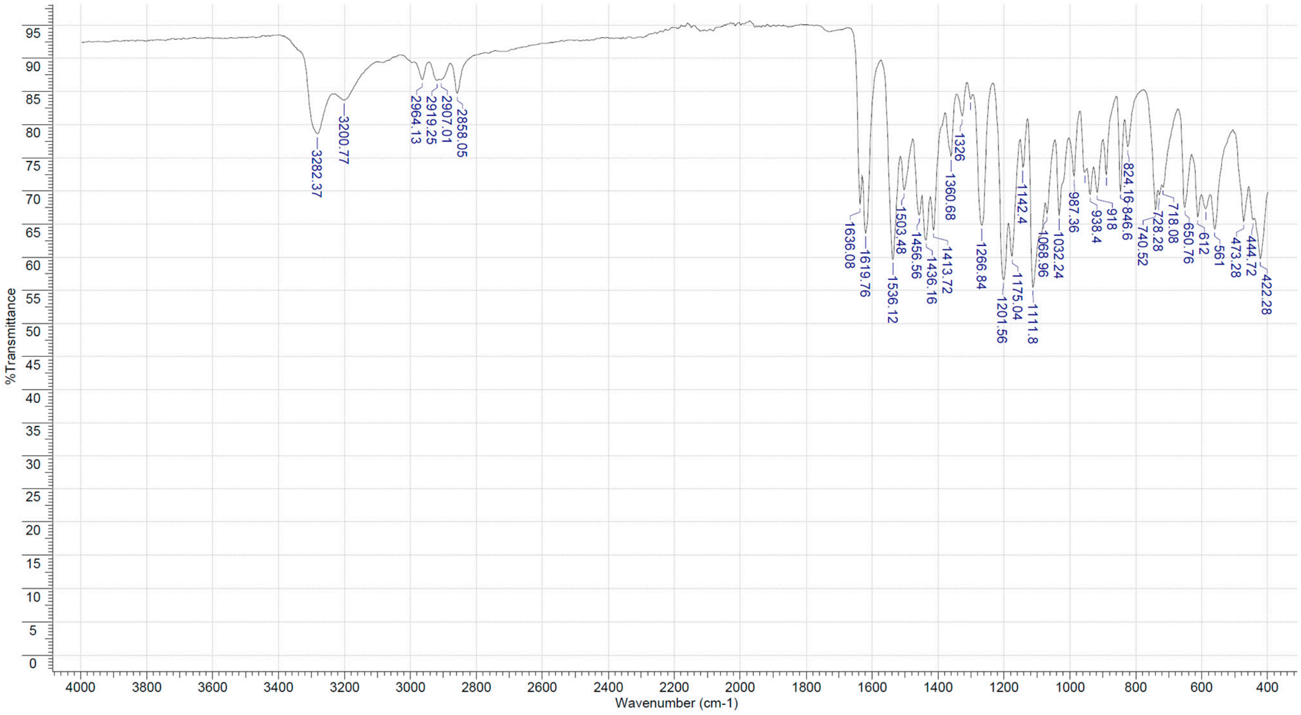

Figure S4. FTIR spectrum of **HL**.

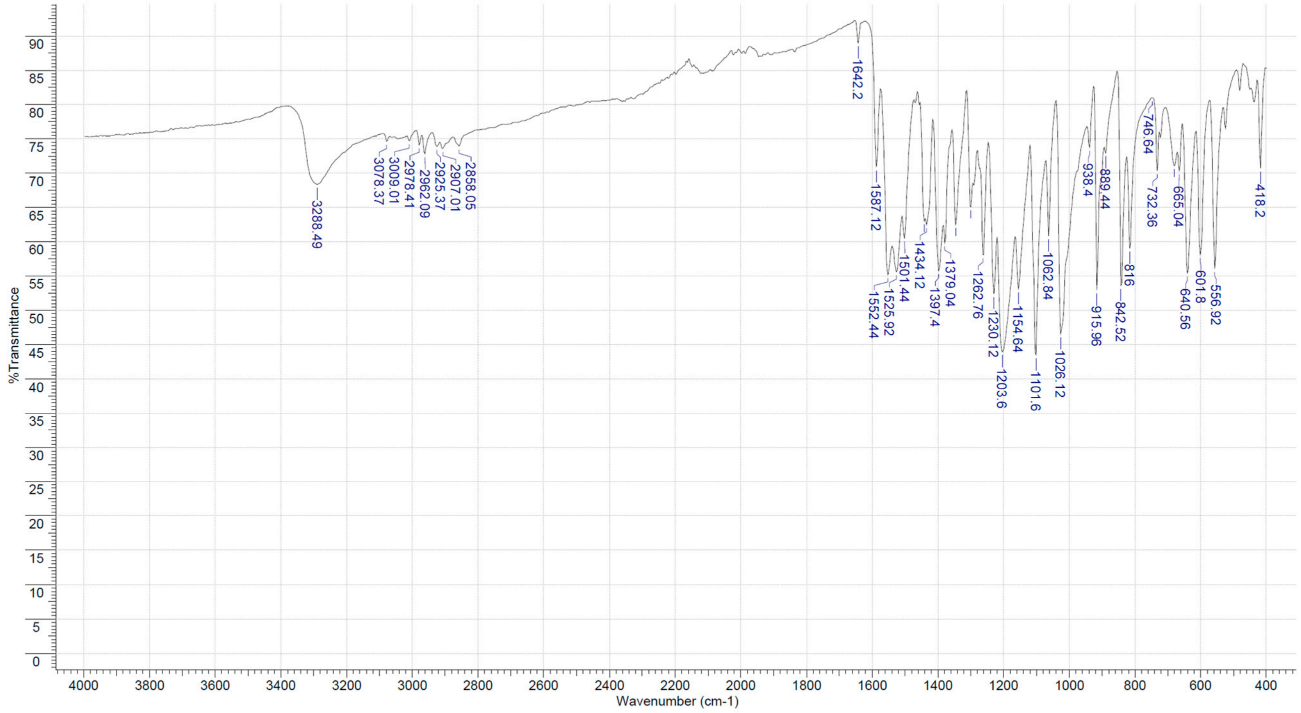

Figure S5. FTIR spectrum of **1**.

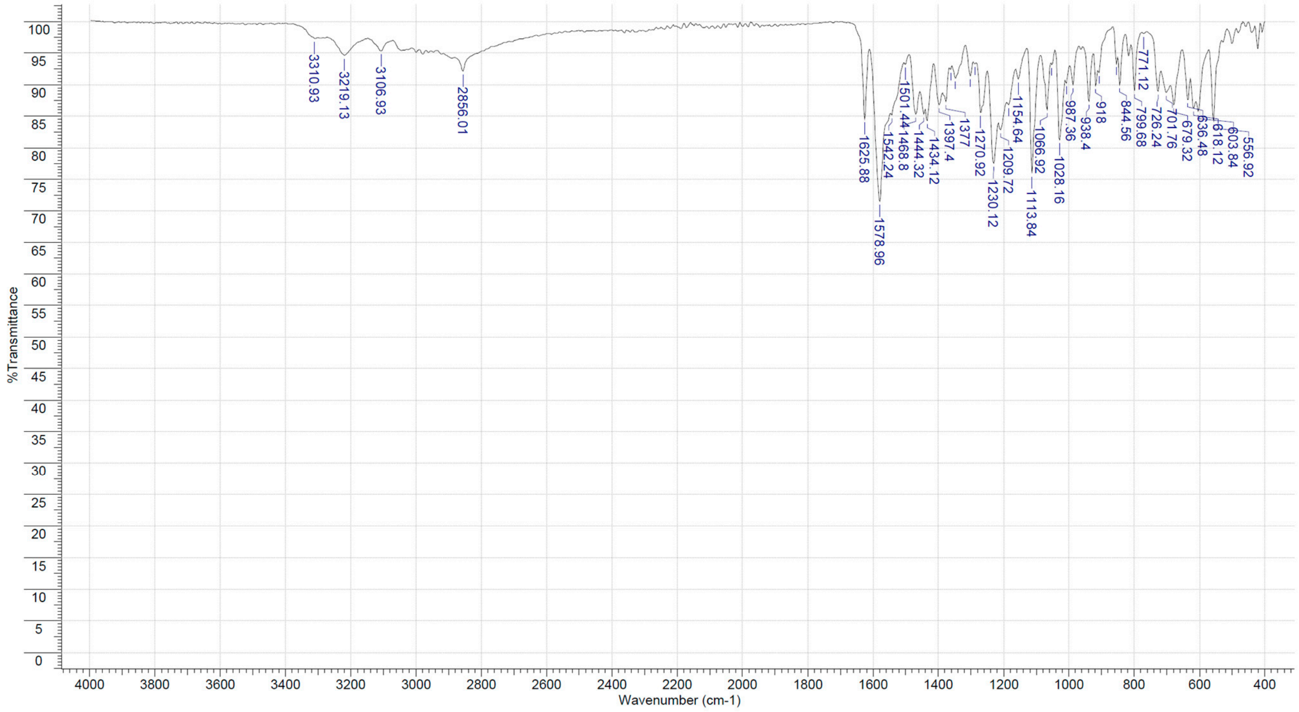

Figure S6. FTIR spectrum of **2**.

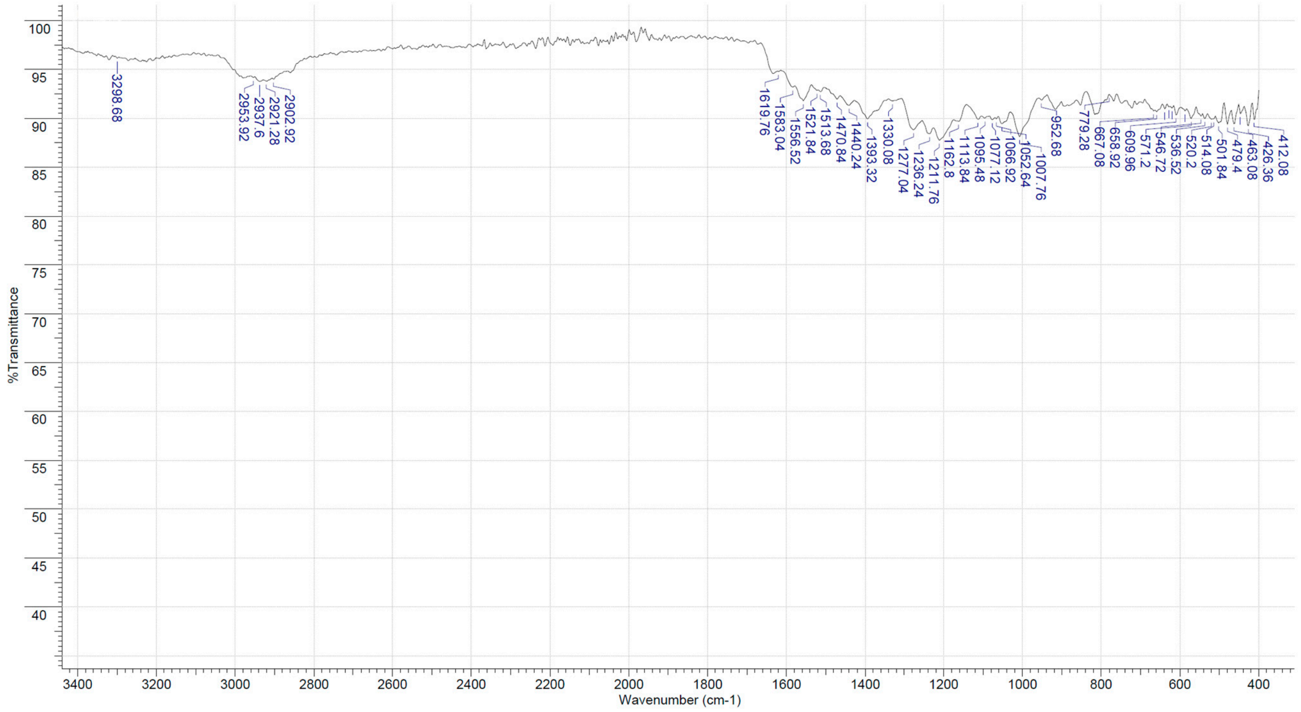

Figure S7. FTIR spectrum of **3**.

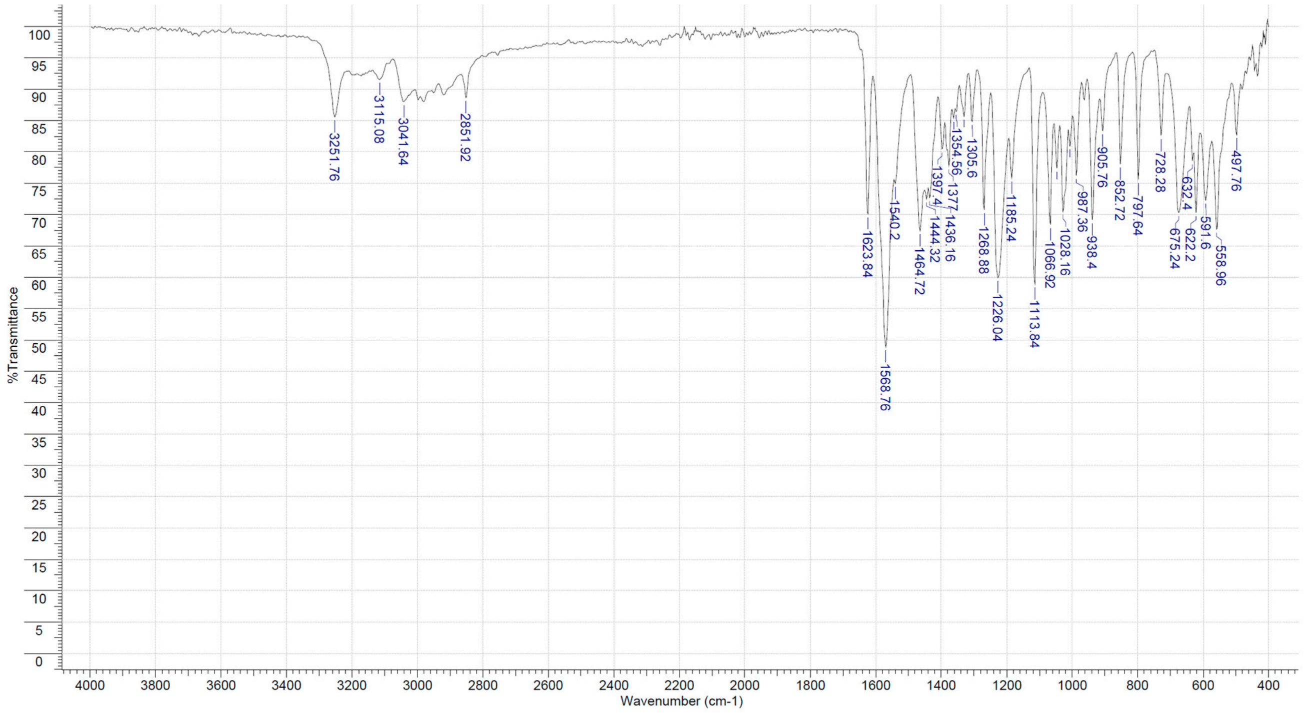

Figure S8. FTIR spectrum of **4**.

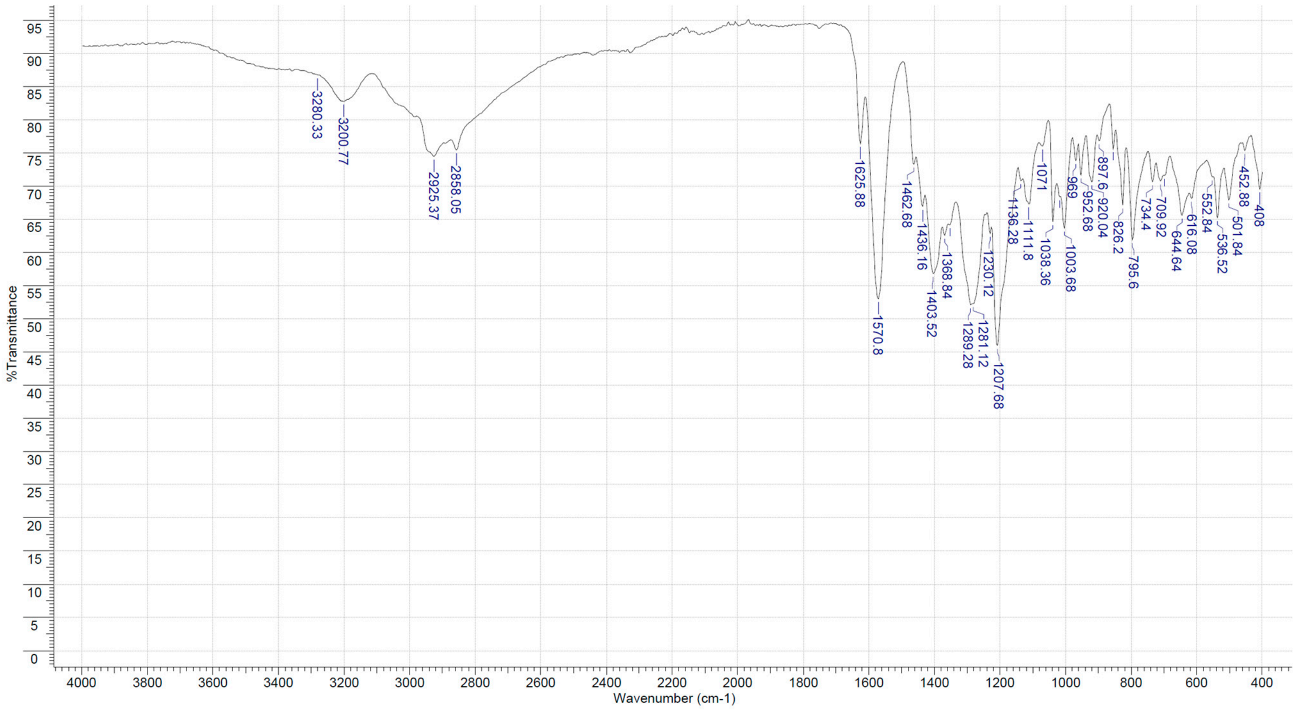

Figure S9. FTIR spectrum of **5**.

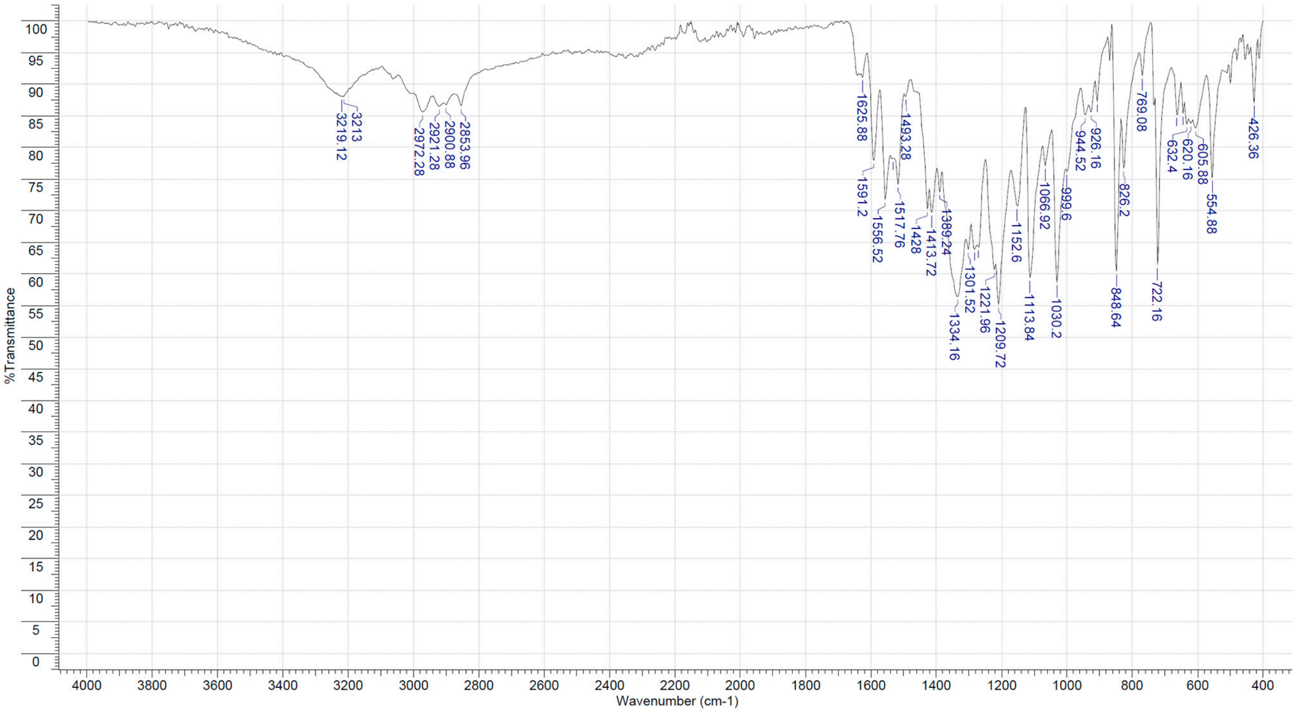

Figure S10. FTIR spectrum of **6**.

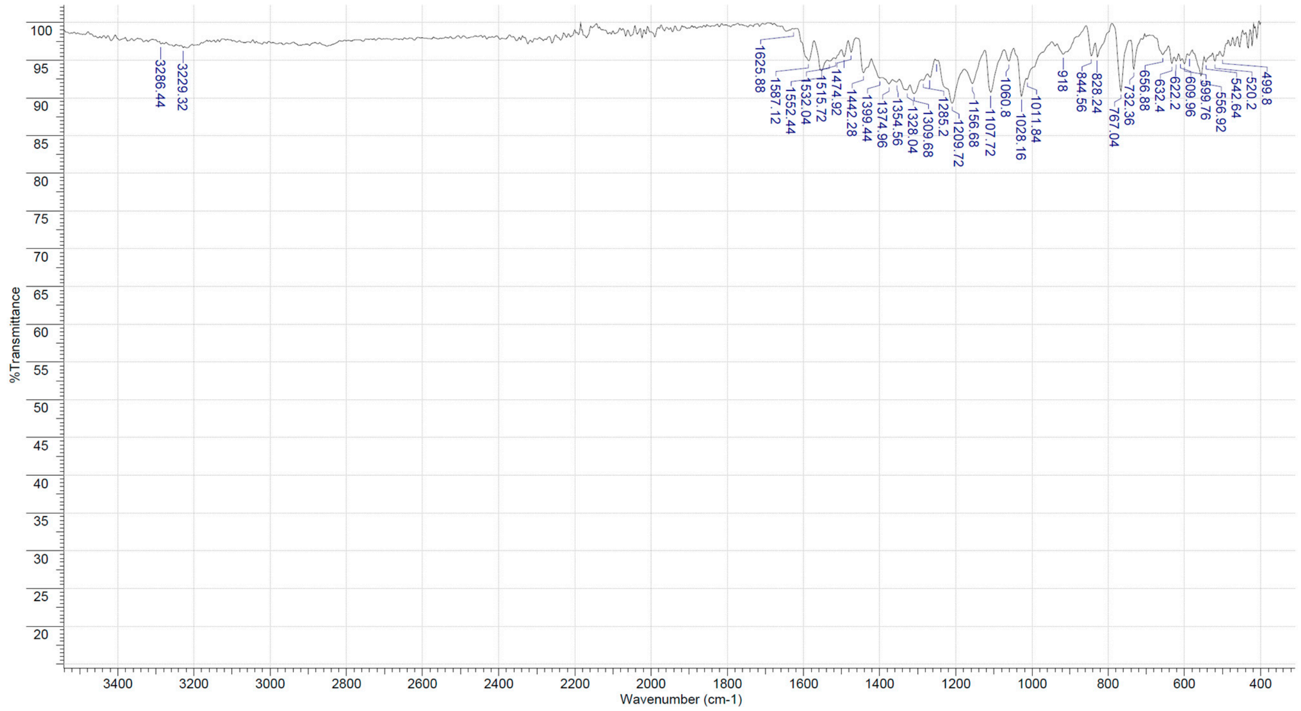

Figure S11. FTIR spectrum of **7**.

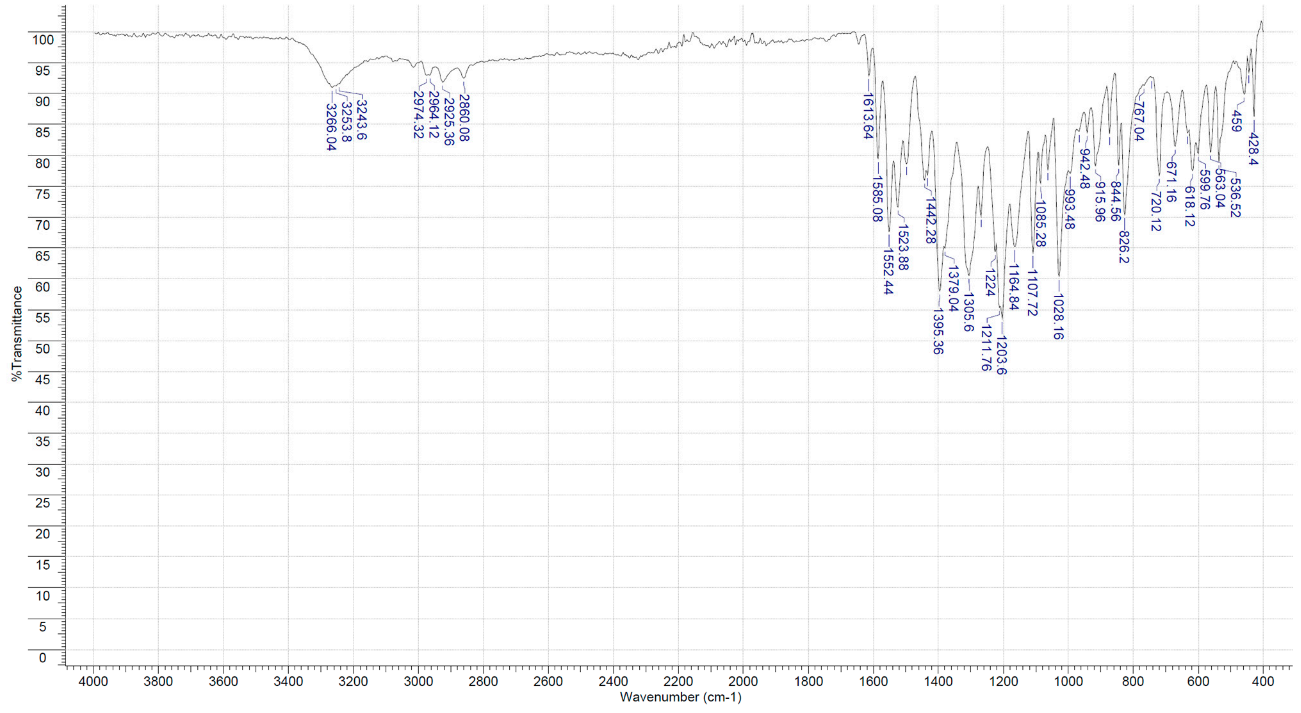

Figure S12. FTIR spectrum of **8**.

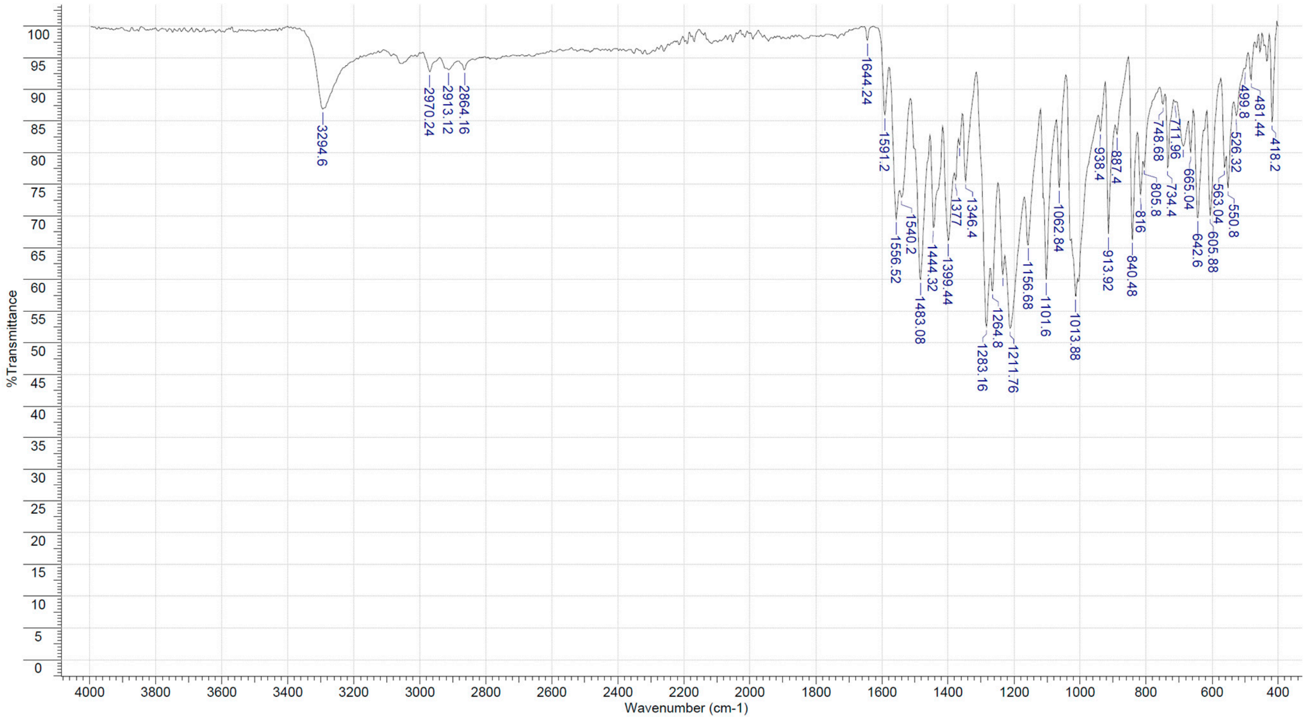

Figure S13. FTIR spectrum of **9**.

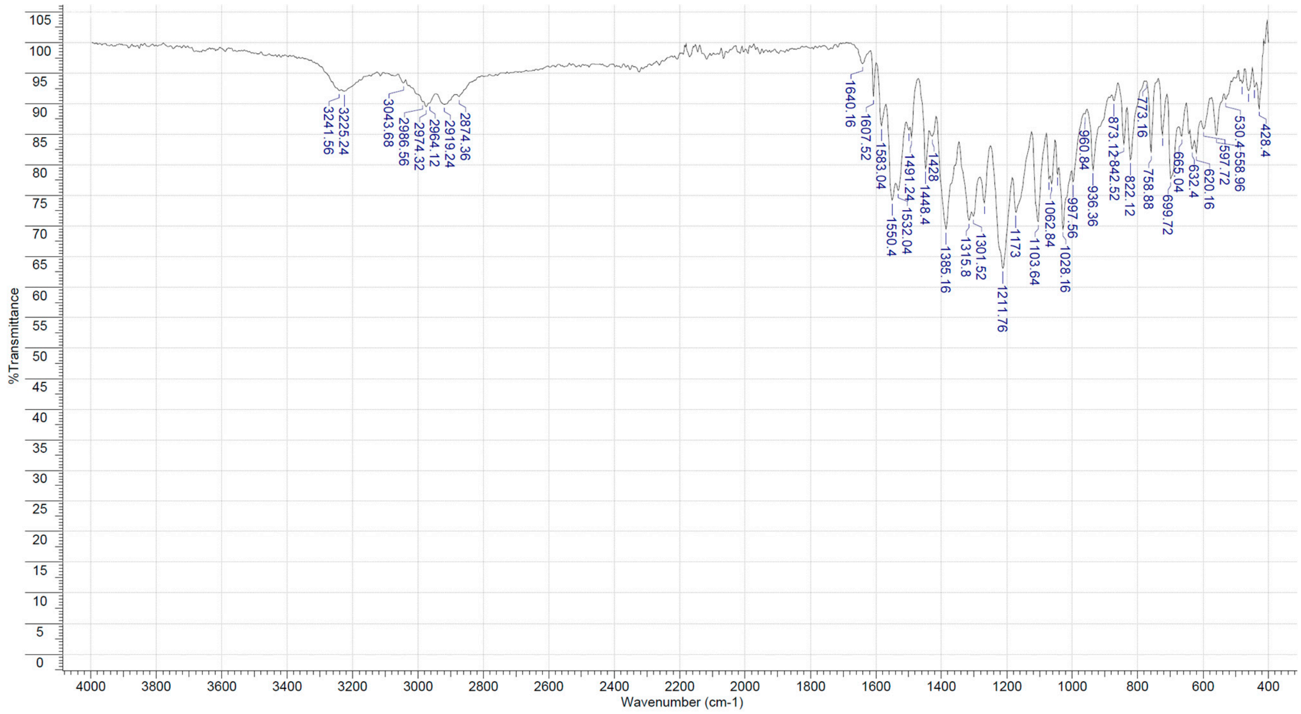

Figure S14. FTIR spectrum of **10**.

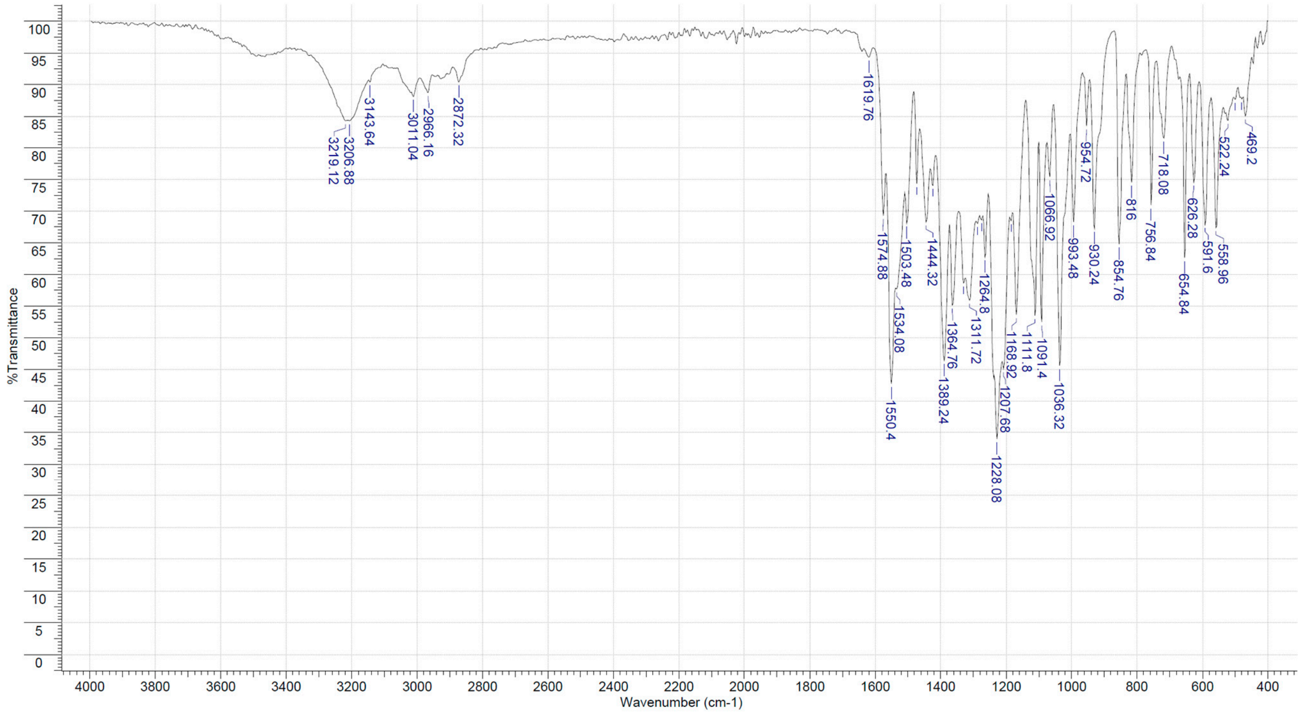

Figure S15. FTIR spectrum of **11**.

Table S1. Crystal and Structure Refinement Date for **HL** and **1, 6, 7,** and **11**

Compound	**HL**	**1**	**6**	**7**	**11**
CCDC no.	2350142	2350143	2350144	2350145	2350146
Empirical formula	$C_{11}H_{18}N_4O_2S_1$	$C_{22}H_{34}Br_2Cu_2N_8O_4S_2$	$C_{23}H_{25}Cu_1N_7O_5S_1$	$C_{21.5}H_{27.5}Cu_1N_7O_{5.75}S_1$	$C_{14}H_{21}Cu_1N_7O_5S_1$
Formula weight	270.35	825.59	575.10	571.60	462.98
Crystal system	orthorhombic	triclinic	monoclinic	triclinic	monoclinic
Space group	*Pbca*	$P\bar{1}$	$P2_1/n$	$P\bar{1}$	$P2_1/c$
Unit cell dimensions					
a (Å)	37.982(3)	8.3254(8)	12.8376(9)	9.0210(8)	8.4112(5)
b (Å)	9.2998(9)	9.2423(7)	15.7485(10)	10.7081(9)	19.3128(12)
c (Å)	15.7832(12)	11.8375(9)	13.2912(9)	14.4944(13)	11.9238(6)
α (°)	90	70.592(7)	90	92.328(7)	90
β (°)	90	76.803(7)	108.569(8)	103.944(8)	92.173(5)
γ (°)	90	65.234(8)	90	90.895(7)	90
V (Å^3)	5575.0(8)	775.84(12)	2547.2(3)	1357.3(2)	1936.3(2)
Z	16	1	4	2	4
ρ_{calc} (g cm^{-3})	1.288	1.767	1.500	1.399	1.588
μ_{Mo} (mm^{-1})	0.233	4.123	0.988	0.928	1.278
F(000)	2308	414	1180	593	956
Crystal size (mm)	0.42x0.22x0.04	0.30x0.11x0.10	0.22x0.16x0.04	0.50x0.30x0.20	0.22x0.16x0.04
θ Range (°)	3.01 – 25.05	2.93 – 25.04	2.97 – 25.05	3.03 – 25.05	3.10 – 25.50
Index range	$-45 \le h \le 26$, $-6 \le k \le 11$, $-18 \le l \le 11$	$-9 \le h \le 6$, $-11 \le k \le 8$, $-14 \le l \le 14$	$-15 \le h \le 8$, $-18 \le k \le 11$, $-15 \le l \le 15$	$-10 \le h \le 10$, $-12 \le k \le 12$, $-17 \le l \le 13$	$-6 \le h \le 10$, $-22 \le k \le 23$, $-13 \le l \le 14$
Reflections collected / unique	13145 / 4910 (R_{int} = 0.0540)	4544 / 2720 (R_{int} = 0.0271)	8947 / 4491 (R_{int} = 0.0540)	8513 / 4786 (R_{int} = 0.0312)	7042 / 3600 (R_{int} = 0.0322)

Reflections with $I > 2\sigma(I)$	2405	2103	2467	3444	2625
Number of refined parameters	353	183	383	345	254
Goodness-of-fit (GOF)	1.003	1.002	1.004	1.004	0.998
R (for $I > 2\sigma(I)$)	R_1 = 0.0828, wR_2 = 0.2014	R_1 = 0.0407, wR_2 = 0.0902	R_1 = 0.0670, wR_2 = 0.1600	R_1 = 0.0635, wR_2 = 0.1864	R_1 = 0.0617, wR_2 = 0.1628
R (for all reflections)	R_1 = 0.1617, wR_2 = 0.2431	R_1 = 0.0595, wR_2 = 0.0989	R_1 = 0.1312, wR_2 = 0.1909	R_1 = 0.0928, wR_2 = 0.2095	R_1 = 0.0853, wR_2 = 0.1788
$\Delta\rho_{max}/\Delta\rho_{min}$ (e·Å^{-3})	0.406 / –0.382	0.762 / –0.607	0.613 / –0.358	0.679 / –0.391	0.657 / –0.496

Table S2. **a)** Bond Lengths (Å) and Angles (deg) in Coordination Metal Environment in **1**, **6**, **7**, and **11**

Bonds	**1**, (Å)	**6**, (Å)	**7**, (Å)	**11**, (Å)
Cu(1)–O(1)	2.023(3)	2.226(4)	2.115(3)	1.978(3)
Cu(1)–N(2)	1.969(3)	1.937(5)	1.938(4)	1.940(4)
Cu(1)–S(1)	2.235(1)	2.255(2)	2.243(1)	2.232(1)
Cu(1)–Br(1)/N(5)	2.3775(6)	1.976(4)	1.972(4)	1.938(4)
Cu(1)–Br(1)*/N(6)	2.9514(8)	2.116(5)	2.147(4)	
Angles	**1**, (°)	**6**, (°)	**7**, (°)	**11**, (°)
O(1)–Cu(1)–N(2)	79.62(13)	76.3(2)	78.6(1)	81.4(1)
O(1)–Cu(1)–S(1)	162.62(8)	134.1(1)	151.4(1)	165.9(1)
O(1)–Cu(1)–Br(1)/N(5)	96.14(8)	98.5(2)	95.4(2)	93.5(2)
O(1)–Cu(1)–Br(1)*/N(6)	82.97(8)	86.5(2)	88.6(1)	
N(2)–Cu(1)–S(1)	84.75(11)	83.9(2)	85.0(1)	85.1(1)
N(2)–Cu(1)–Br(1)/N(5)	168.6(1)	174.8(2)	172.5(2)	174.7(2)
N(2)–Cu(1)–Br(1)*/N(6)	93.6(1)	98.3(2)	104.9(2)	
S(1)–Cu(1)–Br(1)/N(5)	97.76(5)	99.8(1)	98.7(1)	99.9(1)
S(1)–Cu(1)–Br(1)*/N(6)	105.74(5)	139.9(1)	118.4(1)	
Br(1)/N(5)–Cu(1)–Br(1)*/N(6)	96.35(3)	81.2(2)	79.2(2)	

b) Selected Bond Lengths (Å) and Angles (deg) in ligands in **HL**, **1**, **6**, **7**, and **11**

Bonds	**(HL) (A/B)**	**1**	**6**	**7**	**11**
	(Å)				
N(2)–C(6)	1.266(5)/1.287(5)	1.284(5)	1.290(7)	1.300(6)	1.296(5)
N(2)–N(3)	1.367(4)/1.366(5)	1.361(3)	1.365(6)	1.366(5)	1.354(5)
C(8)–N(3)	1.356(3)/1.361(5)	1.327(5)	1.321(7)	1.334(6)	1.319(5)
C(8)–N(4)	1.334(5)/1.311(6)	1.337(5)	1.341(7)	1.340(6)	1.319(6)
C(8)–S(1)	1.666(5)/1.669(5)	1.735(4)	1.734(6)	1.738(5)	1.754(5)
N(4)–C(9)	1.436(6)/1.456(6)	1.437(5)	1.462(8)	1.454(7)	1.473(7)
Angles	(°)				
C(6)–N(2)–N(3)	119.1(3)/117.6(4)	123.1(2)	119.3(5)	118.1(4)	119.3(4)
N(2)–N(3)–C(8)	120.1(3)/119.3(4)	107.0(2)	110.9(5)	110.5(4)	111.7(4)
N(3)–C(8)–N(4)	115.8(4)/116.7(4)	122.9(3)	116.7(6)	117.1(4)	117.9(4)
N(3)–C(8)–S(1)	120.3(3)/117.5(4)	117.0(2)	125.2(5)	125.9(4)	124.8(4)
N(4)–C(8)–S(1)	123.8(4)/125.8(4)	120.1(2)	118.1(5)	117.0(3)	117.3(3)
C(8)–N(4)–C(9)	125.1(4)/124.6(5)	122.1(3)	122.3(6)	123.1(4)	122.6(4)

*-x+2, -y+1, -z

Table S3. Hydrogen Bond Distances (Å) and Angles (deg) for **HL** and **1**, **6**, **7**, and **11**

D–H···A	d(H···A)	d(D···A)	∠(DHA)	Symmetry transformation for acceptor
**HL**				
N(3A)–H(3)···S(1A)	2.68	3.537(4)	171	–*x*+1, –*y*–1, –*z*+1
N(4A)–H(4)···O(1B)	2.10	2.830(5)	142	*x*, –*y*–1/2, *z*+1/2
N(4B)–H(4)···O(1A)	2.23	2.945(5)	142	*x*, *y*, *z*
**1**				
N(4)–H(4)···O(2)	2.11	2.894(5)	152	*x*, *y*, *z*+1
**6**				
N(4)–H(4)···O(3)	2.31	3.17(4)	173	*x*, *y*, *z*
N(4)–H(4)···O(5)	2.11	2.88(2)	149	–*x*+2, –*y*, –*z*
N(4)–H(4)···O(4′)	2.33	3.00(2)	135	–*x*+2, –*y*, –*z*
N(4)–H(4)···O(5′)	2.09	2.95(2)	173	*x*, *y*, *z*
**7**				
N(4)–H(4)···O(4)	2.17	3.022(7)	174	*x*, *y*, *z*
O(1*w*)–H(2)···O(4)	2.08	2.93(2)	164	*x*, *y*, *z*
O(1M)–H(1)···O(3)	2.44	3.02(1)	128	*x*, *y*, *z*
**11**				
N(4)–H(4)···O(4)	2.31	3.038(6)	142	*x*, *y*, *z*
N(6)–H(6)···O(3)	2.41	3.185(8)	150	–*x*+1, *y*–1/2, –*z*+5/2
N(6)–H(6)···O(4)	2.04	2.840(6)	154	–*x*+1, *y*–1/2, –*z*+5/2
